# Experiences of social isolation among older adults with type 2 diabetes mellitus: A qualitative study

**DOI:** 10.1371/journal.pone.0354450

**Published:** 2026-08-10

**Authors:** Shanshan Hu, Chaxiang Li, Wei Zhang

**Affiliations:** 1 Affiliated Central People’s Hospital, China Three Gorges University / Yichang Central People's Hospital, Yichang, Hubei, China; 2 College of Medicine and Health Sciences, China Three Gorges University, Yichang, Hubei, China; University of Porto Faculty of Medicine: Universidade do Porto Faculdade de Medicina, PORTUGAL

## Abstract

**Objective:**

To explore the experiences of social isolation among older adults with type 2 diabetes mellitus and identify the individual, interpersonal, and environmental factors shaping these experiences.

**Design:**

A descriptive qualitative study was conducted. Fourteen older adults with T2DM were recruited from the Department of Endocrinology of a tertiary hospital in Yichang, China, using purposive sampling.

**Methods:**

Face-to-face semi-structured interviews were conducted, and data were analysed using qualitative content analysis.

**Results:**

Three main themes were identified. The first theme, intrapersonal withdrawal, reflected illness-related loss, fear of disease progression and emotional loneliness. The second theme, relational disconnection, described constrained autonomy caused by overprotective family care, shrinking peer networks and insufficient psychosocial support in doctor-patient communication. The third theme, environmental exclusion, involved mismatched community services, inaccessible public spaces and diabetes-related stigma. These themes indicate that social isolation among older adults with T2DM is a multi-level process shaped by the interaction of physical and psychological distress, relational burden and environmental barriers.

**Conclusion:**

Social isolation among older adults with T2DM is not merely a lack of social contact, but a complex experience involving individual vulnerability, weakened interpersonal connections and restricted opportunities for social participation. Healthcare professionals should pay attention to patients’ emotional loneliness, autonomy and social participation needs. Family- and community-based support should also be strengthened to create more inclusive and age-friendly environments for this population.

## 1. Introduction

Type 2 diabetes mellitus (T2DM) is one of the most common chronic metabolic diseases among older adults and is mainly characterized by insulin resistance and progressive pancreatic β-cell dysfunction. With population ageing and lifestyle changes, the burden of T2DM among older adults has become increasingly prominent. In China, diabetes affects approximately 30% of individuals aged 60 years and above, and more than 95% of these cases are type 2 diabetes [1,2]. Older adults with T2DM often require long-term pharmacological treatment, lifestyle modification, blood glucose monitoring and continuous self-management. In addition, age-related functional decline and diabetes-related complications, such as cardiovascular, cerebrovascular, renal and peripheral vascular complications, may further increase their physical, psychological and social burden [3].

For older adults, T2DM is not only a biomedical condition but also a long-term life event that may reshape daily routines, family roles and social participation [4]. Strict dietary control, medication adherence, fear of hypoglycaemia, reduced physical mobility and concerns about complications may limit opportunities for social interaction. Some patients may gradually reduce participation in family, community or recreational activities because of fatigue, physical discomfort, perceived stigma or lack of confidence in managing diabetes outside the home. Over time, these experiences may place older adults with T2DM at increased risk of social isolation.

Social isolation generally refers to insufficient social contacts, limited social networks and reduced engagement in social relationships or social activities. It is conceptually distinct from loneliness, which reflects a subjective feeling of dissatisfaction with one’s social relationships [5,6]. In older adults with chronic illness, social isolation may arise from the interaction of disease-related limitations, psychological distress, reduced mobility, family support changes and environmental barriers [7]. Therefore, social isolation should not be understood simply as an individual’s voluntary withdrawal, but rather as a multidimensional phenomenon shaped by health, psychological, interpersonal and social contexts.Increasing evidence suggests that social isolation is associated with adverse health outcomes in individuals with diabetes. Previous studies have reported that social isolation and loneliness are associated with a higher risk of diabetic microvascular complications, and that these associations may be comparable in importance to several traditional lifestyle-related risk factors [8]. Other research has shown that individuals with diabetes who experience higher levels of social isolation tend to have poorer glycaemic control, including higher fasting blood glucose and glycated haemoglobin levels [9]. These findings indicate that social isolation may be an important and potentially modifiable social determinant of health among people with T2DM.

However, existing research on social isolation in older adults with T2DM remains limited, particularly in the Chinese context. Most available studies have used quantitative designs to examine prevalence, associated factors or health outcomes [10,11]. Although such studies are valuable for identifying statistical associations, they are less able to explain how older patients understand, experience and respond to social isolation in their everyday lives. In particular, little is known about the subjective experiences of older Chinese adults with T2DM, including how disease management, family relationships, community participation, psychological changes and cultural expectations shape their social connections.A qualitative approach is therefore needed to obtain a deeper understanding of the lived experiences and contextual meanings of social isolation among older adults with T2DM [12]. By allowing participants to describe their own experiences in detail, qualitative interviews can reveal the processes through which social isolation develops, the barriers that prevent social participation, and the types of support that patients perceive as meaningful. Such evidence may help healthcare professionals move beyond general risk assessment and develop more patient-centred, culturally appropriate interventions.

Therefore, this study adopted a descriptive qualitative design to explore the experiences of social isolation among older adults with T2DM in China. Through in-depth interviews, this study aimed to understand how these patients perceive social isolation, what factors contribute to their social disconnection, and what forms of support they need. The findings may provide evidence for healthcare professionals to identify socially isolated patients more effectively and to develop targeted interventions to improve social participation, self-management and quality of life among older adults with T2DM.

## 2. Materials and methods

### 2.1. Study design

This study adopted a descriptive qualitative design to explore the experiences of social isolation among older adults with type 2 diabetes mellitus (T2DM). A descriptive qualitative approach was considered appropriate because the purpose of the study was not to test a predetermined theory, but to obtain a direct and detailed understanding of how older patients perceive, experience and respond to social isolation in their everyday lives. The study was guided by the socio-ecological framework, which emphasizes that individual experiences are shaped by interactions among individual, interpersonal, community and broader social contexts. This framework was used to inform the development of the interview guide and the interpretation of the findings. The reporting of this study was guided by the Consolidated Criteria for Reporting Qualitative Research (COREQ).

### 2.2. Participant selection and recruitment

Participants were recruited from the Department of Endocrinology of a tertiary Grade A hospital in Yichang, China, between 10 November 2025 and 31 December 2025. Purposive sampling was used to recruit participants who could provide rich and relevant information about the experience of social isolation in the context of living with T2DM. To improve the diversity of the sample, the research team sought maximum variation in gender, age, marital status, educational level, occupational background and duration of illness.

The inclusion criteria were as follows: 1) diagnosis of T2DM according to the Chinese Guidelines for the Prevention and Treatment of Type 2 Diabetes; 2) age 60 years or older; 3) clear consciousness and ability to communicate verbally; and 4) willingness to participate and provide written informed consent. The exclusion criteria were: 1) diagnosed psychiatric disorder or cognitive impairment; 2) severe hearing, speech or communication difficulties; and 3) refusal or inability to complete the interview.These criteria were used to ensure that participants were able to understand the interview questions and provide rich, reliable narrative data. The diagnosis of T2DM was based on the Chinese Guidelines for the Prevention and Treatment of Type 2 Diabetes, and the reporting of the qualitative study followed the Consolidated Criteria for Reporting Qualitative Research (COREQ) [13].

Potential participants were first identified with the assistance of clinical nurses and medical staff. Eligible patients were then approached by the researcher, who explained the purpose, procedures, voluntary nature and confidentiality of the study. Fourteen participants were finally included. Participants were coded as N1–N14 to protect their identity. Their demographic characteristics are shown in Table 1.

**Table 1:** Demographic and disease-related characteristics of participants (*n* = 14).

Number	Gender	Age (years)	Marital status	Educational attainment	Duration of T2DM (years)
N1	Male	70-79	Married	Technical secondary school	12
N2	Female	70-79	Married	Primary school	8
N3	Male	70-79	Widowed	Primary school	15
N4	Male	70-79	Married	Junior secondary school	11
N5	Female	60-69	Married	College	6
N6	Male	70-79	Married	Junior secondary school	7
N7	Male	70-79	Widowed	Primary school	8
N8	Male	70-79	Widowed	College	10
N9	Female	70-79	Married	Junior secondary school	11
N10	Male	60-69	Married	Technical secondary school	15
N11	Female	60-69	Married	Primary school	13
N12	Female	70-79	Widowed	Primary school	14
N13	Male	60-69	Married	Junior secondary school	16
N14	Female	60-69	Married	Junior secondary school	18

The sample size was determined by information saturation rather than by statistical calculation. Data collection and preliminary analysis were conducted concurrently. After each interview, the research team reviewed the transcript and field notes to determine whether new information, codes or categories emerged. After the twelfth interview, the main categories had become stable; two additional interviews were conducted to confirm saturation. As no new themes emerged from these additional interviews, data collection was stopped after 14 interviews.

The 14 participants were aged 60–79 years, with 9 participants in their 70s and 5 in their 60s. The duration of T2DM ranged from 6 to 18 years, with a mean duration of 11.71 ± 3.65 years.

### 2.3. Development of the interview guide

The semi-structured interview guide was developed based on the socio-ecological framework, a review of relevant literature and repeated discussion among the research team. The guide focused on patients’ physical and psychological experiences after diagnosis, changes in family and peer relationships, interactions with healthcare professionals, participation in community activities, perceived disease-related stigma and support needs.Before formal data collection, three pilot interviews were conducted to assess the clarity, order and appropriateness of the questions. Based on the pilot interviews, several questions were revised to make them more understandable for older adults and to encourage open narration. The data from the pilot interviews were not included in the final analysis. The final interview questions are presented in Table 2.

**Table 2:** Semi-structured interview guide.

Question Number	Interview Questions
1.	What physical and psychological changes have you experienced since being diagnosed with T2DM, and how have these changes affected your social interactions?
2.	Compared with the period before your illness, have there been any changes in the attitudes of your family members, friends or neighbours toward you?
3.	During medical visits, how do healthcare professionals pay attention to your psychological and social needs?
4.	Do you usually participate in community or recreational activities? What factors influence your participation?
5.	Do other people’s perceptions of diabetes affect your willingness to participate in social activities?
6.	What types of support would you like to receive?

### 2.4. Data collection

All interviews were conducted face-to-face in a quiet and private room in the hospital to protect participants’ privacy and reduce external interruptions. The interviews were conducted by a postgraduate nursing student trained in qualitative interviewing, who had received training in qualitative research methods and interview skills. Before the interview, the researcher introduced herself, explained that participation was voluntary and emphasized that refusal or withdrawal would not affect the participant’s medical care.

Each interview lasted approximately 45 minutes. With participants’ permission, all interviews were audio-recorded. During the interviews, the researcher followed the interview guide flexibly, encouraged participants to describe their experiences in their own words, avoided leading questions and recorded non-verbal information such as emotional changes, pauses, facial expressions and gestures in field notes. After each interview, the researcher wrote reflective notes to record initial impressions, contextual information and potential influence of the researcher’s assumptions on data interpretation.

### 2.5. Researcher reflexivity

The research team recognized that researchers’ professional background in nursing and prior knowledge of diabetes care might influence the interview process and interpretation of data. To reduce this influence, the interviewer used open-ended questions, avoided giving evaluative responses and encouraged participants to express both positive and negative experiences. Reflexive notes were written after each interview and discussed within the research team. During data analysis, the researchers repeatedly compared interpretations with original transcripts to ensure that the findings were grounded in participants’ accounts rather than in researchers’ preconceptions.

### 2.6. Data analysis

Audio recordings were transcribed verbatim within 24 hours after each interview. The transcripts were checked against the original recordings to ensure accuracy. Field notes were integrated with the transcripts to provide contextual information. Data were managed using NVivo 12.0 software.

Data were analyzed using qualitative content analysis. First, two researchers independently read the transcripts repeatedly to become familiar with the overall content. Second, meaningful units related to social isolation were identified and assigned initial open codes. Third, codes with similar meanings were compared, merged and organized into subcategories. Fourth, subcategories were further abstracted into broader categories and themes through repeated comparison across participants. Finally, the themes were reviewed and refined by the research team to ensure that they accurately reflected the data.

Disagreements in coding or theme development were resolved through discussion. When consensus could not be reached, a senior qualitative researcher was consulted. An audit trail was maintained, including original transcripts, coding records, memos, theme development notes and meeting records.

### 2.7. Ethical considerations

This study was approved by the Ethics Committee of Yichang Central People’s Hospital (Approval No.2025-364-01). All participants were informed of the study purpose, procedures, potential risks and benefits, confidentiality measures and their right to withdraw at any time without affecting their medical treatment. Written informed consent was obtained from all participants before data collection. Audio recordings, transcripts and field notes were stored securely and accessible only to the research team. Participants’ names were replaced with codes N1–N14 in all transcripts and reports.

## 3. Results

### 3.1. Intrapersonal withdrawal: Illness-related loss, fear and emotional loneliness

#### 3.1.1. Loss of self-worth and reduced social confidence.

Several participants reported that diabetes-related symptoms, functional decline and dependence on others weakened their sense of value. This did not only affect their mood but also changed their social behaviour. Some participants avoided social occasions because they felt they could no longer contribute or feared becoming a burden to others.

*“I used to sing tenor in our community seniors’ choir. Now I only watch from afar—I feel like a burden when I join them.”*(N6)

*“I used to supervise more than twenty workers and could identify machine faults with my eyes closed. Now I feel useless.”*(N1)

Another participant linked physical decline with the loss of social recognition:

*“Neighbours used to ask me to tailor clothes for them. Now my hands go numb, my eyesight is blurred, and they have gradually stopped coming.”*(N2)

*“Before retirement, I managed dozens of workers and made decisions efficiently. Now I feel like an old file forgotten in a warehouse corner, never to be looked at again.” (*N*8)*

#### 3.1.2. Fear of disease progression and future dependence.

Some interviewees described persistent anxiety about disease progression, future care needs, financial security, and personal dignity. Their concerns were not limited to physical complications, but also involved the possibility of becoming dependent on family members or being unable to afford future treatment. This uncertainty became an invisible emotional burden that permeated daily life.

*“Today I fear high blood sugar; tomorrow I fear complications. Living like this… truly feels tasteless.” (N1)*

This anxiety was often triggered by bodily symptoms and fears of diabetes-related complications:

*“I live with constant fear of blood sugar spikes and complications. When my legs go numb, I worry about diabetic foot, and the anxiety follows me even into sleep.” (N10)*

For some participants, illness-related uncertainty was also closely tied to anticipated dependency on their children:

*“My children have their own families; I don’t want to become a burden. But the thought of needing care if my condition worsens deeply distresses me.” (N12)*

Financial concerns further intensified this anxiety:

*“Monthly medication and test fees are already a significant expense. If my condition worsens and costs increase, I fear I may not be able to afford treatment.” (N13)*

#### 3.1.3. Emotional loneliness despite physical presence of others.

Some interviewees received regular visits or daily care, yet their illness experiences, psychological burdens, and emotional needs were rarely listened to with genuine understanding or empathy. Their loneliness was therefore not simply a matter of physical absence, but a form of emotional silence in which their stories remained unheard.

*“This loneliness isn’t about whether people are physically present. My son and daughter-in-law bring my grandson to visit on weekends, and the house becomes lively. But once they leave, the silence seems to echo. I have so much I want to say, but nowhere to say it.” (N8)*

For others, infrequent family visits reinforced the feeling of being left alone in daily life:

*“My children are all busy. If they can visit once a week, that is already good enough. Most of the time, I am alone in this empty house.” (N7)*

Even when practical care was available, participants still felt a lack of emotional companionship:

*“My children hired a caregiver who comes twice a day to prepare meals. She makes sure I don’t fall or go hungry, but what I really want is someone to talk to.” (N3)*

### 3.2. Relational disconnection: constrained autonomy and shrinking social networks

#### 3.2.1. Overprotective family care and loss of autonomy.

Family members’ health anxieties and risk-avoidance concerns often led to excessive protection and control. Although framed as care, such management restricted participants’ daily activities, financial autonomy, and social participation, thereby intensifying the tension between dependence and autonomy.

*“My daughter took away my ID card and pension card, and even calls if I walk outside for more than half an hour.” (N3)*

*“Whenever I want to watch people play chess downstairs, my wife tells me to stay home because of my high blood sugar.” (N10)*

*“I wanted to join a senior citizens’ trip, but my wife declined on my behalf.” (N11)*

*“My son orders my meals, my daughter-in-law sorts my pills, and they even schedule when I go downstairs for sun exposure. This love feels heavy.” (N14)*

#### 3.2.2. Shrinking peer networks and disease-centred interactions.

Some participants described how illness gradually reshaped their social relationships. Previous leisure-based friendships weakened as dietary restrictions, reduced stamina, and health concerns limited their participation, while new interactions increasingly centered on diabetes management.

*“I used to meet old friends once or twice a week. After being diagnosed with diabetes, I could no longer drink or eat greasy food, so they gradually invited me less often.” (N8)*

Others withdrew from previous social activities and turned to disease-related peer groups instead:

*“I used to have regular dance partners, but my stamina could no longer keep up. Later, I joined a diabetes WeChat group where people mostly discussed medications and blood sugar control.” (N14)*

Even long-standing intimate friendships became increasingly medicalized:

*“My best friend and I used to talk for hours. Now she only asks about my blood sugar or whether my foot is numb, and then we fall into awkward silence.” (N12)*

#### 3.2.3. Gaps in supportive clinician–patient communication.

Healthcare professionals’ highly technical and task-oriented communication may overlook patients’ psychological needs, thereby intensifying feelings of helplessness and loneliness.

*“Each follow-up feels like an assembly line: the nurse checks my blood pressure and blood sugar, and the doctor only asks whether I have taken my medication.” (N6)*

*“The doctor tells me to exercise moderately, but never explains what ‘moderate’ means or where I should exercise.” (N12)*

*“Specialist appointments are hard to get, and the room is always crowded. Even if I want to mention poor sleep or loneliness, I feel there is no time or space to talk about it.” (N13)*

### 3.3. Environmental exclusion: inaccessible services, unsafe spaces and stigma

#### 3.3.1. Mismatch between community services and patient needs.

Community-organised activities tend to be generic, short-term, and overly technical, failing to effectively support social participation for this group. They overlook their inner needs and hinder patients from building social connections.

*“The community organizes walking and hill-climbing events, but for people like us with unstable blood sugar and weak legs, that is not exercise—it is a risk.” (N7)*

*“The diabetes lecture was full of general principles. After it ended, everyone left, and there was no time for questions. It felt like ticking off a task.” (N5)*

*“Community notices and registrations are now all on WeChat. I wanted to join a health exercise class, but I could not use the mini-programme, so I had to give up.” (N12)*

#### 3.3.2. Insufficient accessibility in public spaces.

Current spatial designs often fail to adequately consider the specific needs of elderly individuals with chronic conditions. This results in a significant reduction of social interaction opportunities they could otherwise enjoy, due to physical barriers and safety concerns.

*“I rarely take the bus now. The stops are far apart, and waiting too long makes my legs weak. I fear a sudden hypoglycaemic episode.” (N4)*

*“Parks look nice, but the toilets are too far apart. Every visit means calculating how far I am from the toilet.” (N9)*

*“The stairs at the community centre are too steep, and there is no lift. When my blood sugar is unstable, climbing them makes me dizzy.” (N11)*

#### 3.3.3. Diabetes-related stigma and social avoidance.

Misunderstanding, wariness, and low social tolerance led some participants to avoid embarrassment, blame, or being perceived as a burden. To protect themselves from negative judgment, they often chose silence, concealment, or withdrawal in social settings, which further weakened their interpersonal connections and deepened their sense of marginalisation.

*“When neighbours know I have diabetes, they ask whether I used to eat too many sweets, as if the illness were my own fault.” (N2)*

*“Once, when I took out my insulin needle in a park, the person beside me quickly moved away. That look made me feel ashamed.” (N7)*

*“I wanted to watch the neighbourhood dance rehearsal, but the leader worried I might faint and said they could not take responsibility. I felt like a burden and never went back.” (N11)*

*“Because of blurred vision, I move slowly at the supermarket checkout. When someone complained that I was holding things up, I felt like a useless old fool.” (N13)*

## 4. Discussion

This study explored the experience of social isolation among older adults with T2DM from a socio-ecological perspective. The findings indicate that social isolation in this population is not merely a state of living alone or having fewer social contacts, but a cumulative process shaped by illness-related vulnerability, changes in interpersonal relationships and environmental exclusion. The three themes identified in this study—intrapersonal withdrawal, relational disconnection, and environmental exclusion—correspond to the individual, interpersonal, and environmental levels of the socio-ecological framework. These levels interacted with each other: physical decline and fear of complications weakened participants’ confidence in social participation; family overprotection and disease-centred relationships reduced autonomy and narrowed social networks; and unsuitable community services, inaccessible public spaces and disease-related stigma further restricted opportunities for participation.

At the individual level, participants’ social isolation was closely related to the loss of previous roles and self-worth. Many participants described T2DM not only as a chronic metabolic disease, but also as a life event that changed their family roles, community participation and sense of personal value. This finding is consistent with previous studies showing that social isolation is associated with poorer health outcomes among people with diabetes, including increased risk of microvascular complications and poorer glycaemic control [14,15]. However, the present qualitative findings further explain how social isolation may be formed in daily life. For some older adults, withdrawal from social activities was not caused only by physical limitations, but also by fear of becoming a burden, reduced confidence and the feeling that their previous social identity had been weakened. This suggests that social isolation should be understood as both a health-related and identity-related experience.

The findings also show that social isolation and loneliness were intertwined but not identical. Some participants were not physically alone and received daily care from family members or caregivers, but they still lacked meaningful emotional communication. This supports the view that social isolation should not be understood only as an objective reduction in social contact, but also as a weakening of meaningful social connection [16]. Previous studies among older patients with T2DM have mainly examined influencing factors through quantitative methods [17,18], whereas the present study adds experiential evidence by showing that emotional isolation may persist even when instrumental support is available. Therefore, clinical assessment should not focus only on whether patients have caregivers, but should also consider whether they feel understood, respected and socially connected.

At the interpersonal level, family support played an ambivalent role. In the Chinese cultural context, family members are often the main source of care for older adults with chronic illness. However, this study found that family care may become restrictive when it is expressed as excessive protection or control. Some participants reported that their family members limited their outdoor activities, controlled their diet and made decisions on their behalf. Although these behaviours were usually motivated by safety concerns, they could reduce patients’ autonomy and reinforce dependence. This finding is consistent with research in other chronic disease populations suggesting that overprotection may reduce older adults’ social roles and willingness to participate [19]. It also echoes studies showing that empowerment and perceived control are important for reducing social isolation and promoting positive coping [20]. Thus, the key issue is not simply whether family support exists, but whether such support preserves patients’ autonomy.

Peer relationships and healthcare communication also contributed to relational disconnection. Some participants gradually withdrew from previous interest-based social circles and shifted toward disease-centred interactions. Diabetes-related groups or conversations may provide disease information, but they cannot fully replace ordinary social life based on shared interests, emotional exchange and role participation. Similarly, some participants perceived clinical encounters as task-oriented and focused mainly on blood glucose, medication and test results. Their loneliness, fear and social difficulties were rarely discussed. This suggests that disease management should not be limited to biomedical indicators. Health education and diabetes management programmes may improve knowledge and self-management ability [21,22], but they also need to include psychosocial assessment and communication support to address the social consequences of chronic illness.

At the environmental level, this study found that community services, public spaces and digital access shaped patients’ opportunities for social participation. Some community activities were too physically demanding or too short-term to meet the needs of older adults with T2DM. Digital registration systems also created barriers for participants with poor eyesight, low digital literacy or limited family support. This is consistent with research indicating that digital exclusion is associated with social isolation and adverse psychological outcomes among older adults [23]. In addition, inaccessible public spaces, long walking distances, insufficient rest facilities and fear of hypoglycaemia or falling made participants less willing to go out. Previous research has also shown that fear of falling and insufficient social support are related to social isolation among older adults with T2DM [24]. These findings indicate that environmental barriers do not merely accompany social isolation; they actively produce and reinforce it.

Disease-related stigma further intensified environmental exclusion. Some participants felt blamed because diabetes was regarded by others as a consequence of poor self-control. Others experienced embarrassment when injecting insulin or moving slowly in public. Such experiences encouraged concealment and avoidance. Previous studies have identified shame and stigma as important factors associated with social isolation among patients with T2DM and other chronic conditions [25,26]. The present study extends these findings by showing that stigma operates not only as an external social judgement but also as an internalised pressure that shapes behaviour. For some participants, social withdrawal was a way to avoid embarrassment, blame or being regarded as a burden.

Overall, this study contributes to the understanding of social isolation among older adults with T2DM by showing that it is produced through the interaction of individual withdrawal, relational disconnection and environmental exclusion. This interpretation moves beyond a simple risk-factor model and highlights the multi-level nature of social isolation. Therefore, interventions should not focus only on encouraging patients to participate in activities. Healthcare professionals should also assess patients’ emotional loneliness, perceived autonomy and social participation needs. Families should be guided to provide autonomy-supportive care rather than excessive protection. Communities should develop age-friendly and diabetes-sensitive services, including low-intensity activities, accessible registration methods and safe public spaces. Public education is also needed to reduce moralising misconceptions about diabetes and promote a more supportive social environment [27].

This study has several limitations. First, participants were recruited from a single tertiary hospital in one city, which may limit the transferability of the findings to older adults with T2DM in other regions, rural communities or primary care settings. Second, although purposive sampling was used and data collection was guided by information saturation, the sample size was relatively small. Third, because the study was based on one-time interviews, it could not capture how experiences of social isolation change over time with disease progression, family changes or community support. Future studies could use multi-centre sampling, include family caregivers and healthcare professionals, and adopt longitudinal or mixed-method designs to further examine the dynamic mechanisms of social isolation among older adults with T2DM.

## 5. Conclusion

This study found that social isolation among older adults with T2DM is a multi-level experience shaped by intrapersonal withdrawal, relational disconnection, and environmental exclusion. Rather than reflecting only a lack of social contact, social isolation involved illness-related vulnerability, weakened interpersonal connections, and restricted opportunities for social participation. Healthcare professionals should attend to patients’ emotional loneliness, autonomy, and participation needs, while families and communities should provide more supportive, inclusive, and age-friendly environments. Future multi-centre and longitudinal studies are needed to further examine the dynamic mechanisms of social isolation and develop targeted interventions for this population.

## Supporting information

S1 File Supplementary materials: Interview transcripts. (DOC)
