# Supplementary material for: Experiences of social isolation among older adults with type 2 diabetes mellitus: A qualitative study
Source: PLoS One. 2026 Aug 10;21(8):e0354450. doi: 10.1371/journal.pone.0354450 (PMC13456196; doi:10.1371/journal.pone.0354450)
Supplement: Supplementary file 4 [file pone.0354450.s006.docx]

**Dear Editor and Reviewers,**

**Thank you very much for your careful evaluation of our manuscript entitled “Qualitative Study on Social Isolation Experience of Elderly Patients with Type 2 Diabetes Mellitus.” We sincerely appreciate the time and effort you have devoted to reviewing our work and providing valuable comments. Your constructive suggestions have been extremely helpful in improving the quality of our manuscript.**

**We have carefully considered all comments and revised the manuscript accordingly. Please find our point-by-point responses below.**

Your consideration is highly appreciated.

Yours sincerely,

On behalf of all authors

**Detailed responses to Reviewer 1**

**Reviewer #1:** The manuscript addresses an important topic but shows consistent weaknesses across all sections. The introduction lacks clarity, logical flow, and a clearly articulated research gap with several language and structural issues. The methodology provides a basic qualitative framework but lacks rigor with insufficient detail on sampling, data saturation, analytical procedures, and trustworthiness criteria. The results are well-organized and supported by rich participant quotes, yet remain largely descriptive with overlap between themes and limited analytical depth. The discussion aligns broadly with the findings but mainly restates results, overemphasizes recommendations and lacks critical engagement with literature and study limitations. Overall, the study requires substantial revision to improve coherence, methodological transparency and analytical rigor. I have provided few points below to improve the article for future correspondence. But this time I am unable to accept its publication due to the standard of the esteem journal.
Thank you very much for your careful and comprehensive evaluation of our manuscript. We sincerely appreciate your constructive comments and fully acknowledge the weaknesses identified in the previous version, including insufficient clarity in the Introduction, limited methodological transparency, descriptive presentation of the Results, and inadequate analytical depth in the Discussion.

**Response：**In response to your comments, we have substantially revised the manuscript throughout. Specifically, we rewrote the Introduction to improve its logical flow and to clarify the research gap and rationale for using a qualitative approach. We expanded the Methods section by specifying the descriptive qualitative design, purposive sampling strategy, participant recruitment, interview procedures, data analysis process, researcher reflexivity, and ethical considerations. We also revised the Results section by reorganizing the themes around the socio-ecological framework and clarifying how each theme reflects the experience of social isolation among older adults with T2DM. In addition, we shortened and refined participant quotations to improve readability and analytical focus. The Discussion section was also revised to move beyond restating findings, with greater emphasis on interpretation, comparison with existing literature, theoretical implications, practical relevance, and study limitations.

We are grateful for the reviewer’s comments, which have helped us substantially improve the coherence, methodological rigor, and analytical quality of the manuscript. We hope that the revised version addresses the concerns raised and better meets the standards of the journal.

Introduction:
The introduction addresses a relevant and important topic (T2DM, ageing, and social isolation). However, it has major issues in clarity, structure, language and logical flow. It currently reads as fragmented and partially incomplete and requires substantial revision before being suitable for publication. It needs deep background check.
• Lack of clarity and coherence: The introduction contains grammatical errors, incomplete sentences and unclear phrasing, making it difficult to follow.

**Response：**Thank you for pointing this out. We have carefully revised the language throughout the Introduction to improve grammatical accuracy, sentence completeness and readability. Fragmented expressions were rewritten into coherent paragraphs, and ambiguous wording was clarified. We also revised several overly direct causal statements into more cautious academic expressions, such as “may limit opportunities for social interaction” and “may place older adults with T2DM at increased risk of social isolation.”

• Poor structure and organization: Ideas are presented in a disjointed manner, with no logical progression from background to research gap and study aim.

**Response：**We agree with this comment. The Introduction has been reorganized to follow a clearer logical structure. The revised version first introduces the burden of T2DM among older adults, then explains how T2DM may affect daily life, roles and social participation. It then defines social isolation and distinguishes it from loneliness. After that, we summarize existing evidence on the association between social isolation and diabetes-related outcomes, identify the limitations of previous quantitative studies, and finally present the rationale and aim of the present qualitative study.

• Weak research gap and justification: The need for the study- especially the qualitative approach is not clearly or convincingly explained.

**Response:**Thank you for this helpful suggestion. We have strengthened the research gap by emphasizing that most existing studies on social isolation among older adults with T2DM have used quantitative designs to examine prevalence, associated factors or health outcomes. However, these studies are less able to explain how older patients understand, experience and respond to social isolation in daily life. We therefore added a clearer justification for using a descriptive qualitative approach, highlighting that qualitative interviews can reveal the processes through which social isolation develops, the barriers to social participation, and the support needs perceived by patients. “

However, existing research on social isolation in older adults with T2DM remains limited, particularly in the Chinese context. Most available studies have used quantitative designs to examine prevalence, associated factors or health outcomes [7,8]. Although such studies are valuable for identifying statistical associations, they are less able to explain how older patients understand, experience and respond to social isolation in their everyday lives. In particular, little is known about the subjective experiences of older Chinese adults with T2DM, including how disease management, family relationships, community participation, psychological changes and cultural expectations shape their social connections.A qualitative approach is therefore needed to obtain a deeper understanding of the lived experiences and contextual meanings of social isolation among older adults with T2DM”

• Inadequate use of literature: Citations are poorly integrated and some claims are oversimplified or lack proper contextual explanation.

**Response：**We have revised the literature integration in the Introduction. Instead of listing findings separately, we now connect the literature to the development of the argument. For example, evidence on diabetic microvascular complications and glycaemic control is used to support the health relevance of social isolation among people with T2DM. We also added contextual explanation to clarify why these findings indicate that social isolation may be an important and potentially modifiable social determinant of health in this population.

“Previous studies have reported that social isolation and loneliness are associated with a higher risk of diabetic microvascular complications, and that these associations may be comparable in importance to several traditional lifestyle-related risk factors [5]. Other research has shown that individuals with diabetes who experience higher levels of social isolation tend to have poorer glycaemic control, including higher fasting blood glucose and glycated haemoglobin levels [6]. These findings indicate that social isolation may be an important and potentially modifiable social determinant of health among people with T2DM.”

• Conceptual and scientific issues: Definitions (e.g., social isolation) are somewhat inaccurate or oversimplified and causal relationships are presented too simplistically.

**Response：**We appreciate this important comment. We revised the definition of social isolation to avoid describing it simply as voluntary self-withdrawal. In the revised manuscript, social isolation is defined as insufficient social contacts, limited social networks and reduced engagement in social relationships or social activities. We also clarified its distinction from loneliness, which reflects a subjective feeling of dissatisfaction with one’s social relationships. In addition, causal relationships were softened throughout the Introduction. We now use cautious language such as “may,” “is associated with,” and “may place older adults at increased risk,” rather than implying direct or deterministic causality.

“Social isolation generally refers to insufficient social contacts, limited social networks and reduced engagement in social relationships or social activities. It is conceptually distinct from loneliness, which reflects a subjective feeling of dissatisfaction with one’s social relationships [3,4]. In older adults with chronic illness, social isolation may arise from the interaction of disease-related limitations, psychological distress, reduced mobility, family support changes and environmental barriers. Therefore, social isolation should not be understood simply as an individual’s voluntary withdrawal, but rather as a multidimensional phenomenon shaped by health, psychological, interpersonal and social contexts.Increasing evidence suggests that social isolation is associated with adverse health outcomes in individuals with diabetes.”

• Language and readability problems: The text requires substantial editing for grammar, conciseness and academic tone.
**Response：**We have substantially edited the Introduction for grammar, conciseness and academic tone. Repetitive and informal expressions were removed, and the section was rewritten in a more coherent and scholarly style. The revised Introduction now presents the background, concept, evidence, research gap and study aim in a more concise and readable manner.

Methodology:
Methodology demonstrates a basic qualitative framework; however, it lacks sufficient rigor, transparency and detail. Key aspects such as sampling justification, data saturation, analytic procedures and trustworthiness criteria are inadequately addressed. In tables no mention of units i.e. Age (years/months/days???), Duration of illness (years/months/days???). These are very basic. Substantial revision is required to meet qualitative research reporting standards.

**Response：**Thank you for this important and detailed comment. We fully agree that the previous version of the Methods section lacked sufficient methodological detail and transparency. In the revised manuscript, we have substantially expanded the Methods section to improve compliance with qualitative research reporting standards. Specifically, we clarified the descriptive qualitative design and its rationale, described the purposive sampling strategy and recruitment procedures in greater detail, added an explanation of sample diversity and data saturation, expanded the description of interview procedures, interviewer role and researcher reflexivity, clarified the coding and theme development process, added strategies to ensure credibility, dependability, confirmability and transferability, reported ethical considerations, and revised Table 1 to specify the units for age and duration of illness.

• Unclear study design: The qualitative approach is not well specified or justified and the choice of design is inadequately explained.

**Response：**Thank you for pointing this out. We have clarified that this study used a descriptive qualitative design. We also added a rationale explaining that this approach was appropriate because the purpose of the study was to obtain a direct and detailed understanding of how older adults with T2DM perceive, experience and respond to social isolation in daily life, rather than to test a predetermined theory. In addition, we stated that the study was guided by the socio-ecological framework and reported according to the Consolidated Criteria for Reporting Qualitative Research (COREQ). In the revised manuscript, the Methods section now explicitly states that the socio-ecological framework informed both the interview guide and the interpretation of the findings.

Revision made in the manuscript: “This study adopted a descriptive qualitative design to explore the experiences of social isolation among older adults with type 2 diabetes mellitus (T2DM). A descriptive qualitative approach was considered appropriate because the purpose of the study was not to test a predetermined theory, but to obtain a direct and detailed understanding of how older patients perceive, experience and respond to social isolation in their everyday lives. The study was guided by the socio-ecological framework, which emphasizes that individual experiences are shaped by interactions among individual, interpersonal, community and broader social contexts. This framework was used to inform the development of the interview guide and the interpretation of the findings. The reporting of this study was guided by the Consolidated Criteria for Reporting Qualitative Research (COREQ).”

• Sampling limitations: Purposive sampling is mentioned but recruitment procedures, sampling rationale, and evidence of data saturation are insufficiently described; single-site sampling limits generalizability.

**Response：**We agree with this comment. We have expanded the description of sampling and recruitment. We clarified that participants were recruited from the Department of Endocrinology of a tertiary Grade A hospital in Yichang, China, and that purposive sampling was used to recruit participants who could provide rich information about social isolation in the context of T2DM. We also added that maximum variation was sought in gender, age, marital status, educational level, occupational background and duration of illness to increase the diversity of perspectives. The single-site design is now acknowledged as a limitation in the Discussion. Table 1 has been revised to include the units “Age (years)” and “Duration of illness (years).”

Revision made in the manuscript: “Participants were recruited from the Department of Endocrinology of a tertiary Grade A hospital in Yichang, China, between 10 November 2025 and 31 December 2025. Purposive sampling was used to recruit participants who could provide rich and relevant information about the experience of social isolation in the context of living with T2DM. To improve the diversity of the sample, the research team sought maximum variation in gender, age, marital status, educational level, occupational background and duration of illness.”

Added text on data saturation:

“The sample size was determined by information saturation rather than by statistical calculation. Data collection and preliminary analysis were conducted concurrently. After each interview, the research team reviewed the transcript and field notes to determine whether new information, codes or categories emerged. After the twelfth interview, the main categories had become stable; two additional interviews were conducted to confirm saturation. As no new themes emerged from these additional interviews, data collection was stopped after 14 interviews.”

Table revision:

“Age” was revised to “Age (years)” and “Duration of illness” was revised to “Duration of illness (years).”

• Insufficient detail in data collection: Interview procedures lack depth with no discussion of researcher reflexivity, interviewer role or adequacy of interview duration.

**Response：**We have expanded the data collection section by specifying where and how interviews were conducted, who conducted the interviews, the interviewer’s qualitative training, the approximate interview duration, the use of audio recording, the use of field notes, and strategies to reduce leading questions. We also added a separate section on researcher reflexivity. The revised manuscript now states that interviews were conducted by a postgraduate nursing student trained in qualitative interviewing and that each interview lasted approximately 45 minutes.

“All interviews were conducted face-to-face in a quiet and private room in the hospital to protect participants’ privacy and reduce external interruptions. The interviews were conducted by a postgraduate nursing student trained in qualitative interviewing, who had received training in qualitative research methods and interview skills. Before the interview, the researcher introduced herself, explained that participation was voluntary and emphasized that refusal or withdrawal would not affect the participant’s medical care. Each interview lasted approximately 45 minutes. With participants’ permission, all interviews were audio-recorded. During the interviews, the researcher followed the interview guide flexibly, encouraged participants to describe their experiences in their own words, avoided leading questions and recorded non-verbal information such as emotional changes, pauses, facial expressions and gestures in field notes. After each interview, the researcher wrote reflective notes to record initial impressions, contextual information and potential influence of the researcher’s assumptions on data interpretation.”

• Weak rigor and quality assurance: Key criteria for qualitative rigor (credibility, dependability, confirmability, transferability) are not addressed.

**Response：**We added a new subsection entitled “Trustworthiness” and described the specific strategies used to enhance credibility, dependability, confirmability and transferability. These included maximum-variation purposive sampling, verbatim transcription, repeated comparison with original data, independent coding by two researchers, team discussion, reflexive notes, an audit trail, and detailed description of the research context and participant characteristics.

“Several strategies were used to enhance the trustworthiness of the study. Credibility was strengthened through purposive maximum-variation sampling, verbatim transcription, repeated reading of transcripts and comparison between codes and original data. During interviews, participants were encouraged to provide concrete examples to support their accounts. Dependability was enhanced by using a semi-structured interview guide, conducting interviewer training and maintaining detailed records of data collection and analysis procedures. Confirmability was supported through independent coding by two researchers, team discussion, reflexive notes and an audit trail that included original transcripts, coding records, memos and theme development notes. Transferability was improved by providing detailed descriptions of the research setting, participant characteristics and contextual conditions, enabling readers to judge the applicability of the findings to other settings.”

• Inadequate data analysis description: The coding and thematic analysis process lacks transparency, with no detail on coding framework, inter-coder agreement, or theme development.

**Response：**Thank you for this comment. We have revised the data analysis section to clarify the analytic steps. We stated that the audio recordings were transcribed verbatim within 24 hours, checked against the original recordings, and managed using NVivo 12.0. We also described the process of repeated reading, identifying meaning units, open coding, grouping similar codes into subcategories, abstracting subcategories into themes, and refining themes through team discussion. We clarified that two researchers independently coded the transcripts and that disagreements were resolved through discussion or consultation with a senior qualitative researcher.

• Ethical and reporting gaps: Ethics approval is not reported and the methodology does not adhere to established qualitative reporting standards.

**Response：**Thank you for highlighting this issue. We have added an ethics statement to the Methods section and stated that written informed consent was obtained from all participants. We also clarified that participant identities were protected using codes N1–N14 and that audio recordings, transcripts and field notes were stored securely. In addition, we added that the study reporting was guided by COREQ.

Results:

Results part partially align with the study objective of exploring social isolation among elderly patients with type 2 diabetes, as they present relevant experiences related to psychological distress, relational dynamics and environmental barriers. However, this alignment is not consistently strong or explicit. The central concept of social isolation is not systematically integrated across all themes and some findings (e.g., environmental or healthcare system issues) appear indirectly related rather than clearly tied to the core objective. Additionally, the lack of deeper analytical interpretation and weak link to the guiding theoretical framework limit the extent to which the findings fully address and advance the stated research aim. There is overlap between themes, limited integration with the theoretical framework and issues with redundancy and verbosity. Greater clarity, conceptual refinement, and analytical interpretation are required to strengthen the findings.

**Response：**Thank you for this detailed and constructive comment. We agree that the previous Results section was overly descriptive and that the relationship between the findings, the core concept of social isolation, and the socio-ecological framework was not sufficiently explicit. In the revised manuscript, we reorganized and refined the Results section to better align with the study objective. Specifically, we clarified that social isolation among older adults with T2DM emerged as a multi-level process involving individual withdrawal, relational disconnection and environmental exclusion. We revised the thematic structure to reduce overlap, added analytical interpretation before and after participant quotations, shortened repetitive quotations, and strengthened the link between each theme and the socio-ecological framework. We also added an integrative paragraph to explain how the three themes jointly reflect the process of social isolation.

• Thematic structure issues: Although themes and subthemes are clearly presented, there is overlap between categories and insufficient justification for the thematic framework.

**Response：**Thank you for this comment. We retained the original three-theme structure because it was consistent with the socio-ecological framework and the study objective. To improve clarity, we added explanatory sentences at the beginning of the Results section to justify the thematic framework. The revised text now clarifies that the three themes correspond to individual, interpersonal and environmental levels of social isolation.

“The analysis showed that social isolation among older adults with T2DM was not merely reflected in living alone or having fewer social contacts. Rather, it emerged as a multi-level process shaped by illness-related vulnerability, altered social relationships and exclusionary environments. The three themes—intrapersonal withdrawal, relational disconnection and environmental exclusion—corresponded to the individual, interpersonal and environmental levels of the socio-ecological framework.”

• Limited analytical depth: The findings are largely descriptive with minimal interpretation or higher-level conceptual analysis.

**Response：**In the revised manuscript, we added short analytical explanations after key participant quotations to clarify how the reported experiences contributed to social isolation. These additions were made without changing the original findings or the participants’ meanings. For example, after quotations describing reduced self-worth, we added an interpretation explaining that loss of perceived usefulness may weaken social confidence and lead to withdrawal from previous social roles.

“These accounts suggest that social isolation was partly rooted in a weakened sense of usefulness. When participants perceived themselves as less capable or as a burden to others, they were more likely to withdraw from previous family, occupational and community roles.”

• Quotation overuse: Participant quotes are rich but often too lengthy and repetitive, reducing clarity and impact. Please learn how quotes are presented in academic journal. Very poor presentation.

**Response：**Thank you for this suggestion. We carefully reviewed the participant quotations and retained those that most directly supported each subtheme. To preserve the authenticity of participants’ voices, we did not substantially change the original meaning of the quotations. However, we improved their presentation by placing them within clearer analytical context and reducing unnecessary repetition in the surrounding explanatory text.

• Weak integration with theory and objectives: The results are not consistently linked to the stated theoretical framework (social ecological theory) or clearly anchored to the core concept of social isolation.

**Response：**We have strengthened the link between the Results section, the study objective and the socio-ecological framework. In the revised manuscript, each main theme is explicitly connected to one level of the socio-ecological framework: individual-level withdrawal, interpersonal-level disconnection and environmental-level exclusion. This revision helps clarify how the findings address the objective of exploring social isolation among older adults with T2DM.

• Redundancy and lack of rigor: Repetition of similar ideas, absence of thematic prevalence indicators and limited transparency in how findings were derived weaken the rigor.

**Response：**Thank you for this helpful comment. We reduced repetitive wording in the Results section and added qualitative prevalence indicators, such as “most participants,” “several participants” and “some participants,” to improve transparency while remaining appropriate for qualitative reporting. We also added an integrative paragraph to show how the themes were derived from participants’ accounts and how they jointly explain the process of social isolation.
“Taken together, the findings suggest that social isolation among older adults with T2DM is produced through the interaction of individual, relational and environmental mechanisms. Illness-related loss and fear weakened motivation for social participation; overprotection, disease-centred relationships and limited psychosocial communication narrowed meaningful social connections; and unsuitable services, inaccessible spaces and stigma further restricted opportunities for participation. As a result, social isolation became a cumulative process of withdrawal, disconnection and exclusion.”

Discussion:

Discussion demonstrates a clear effort to organise findings within a socio-ecological framework and maintains thematic consistency with the results. However, it falls short in delivering critical, in-depth interpretation and scholarly engagement. The section reads more as a set of practical recommendations than a rigorous academic discussion, with insufficient attention to limitations, contextualization and theoretical advancement. Substantial revision is needed to strengthen analytical depth, balance, and scientific contribution.

**Response：**Thank you for this constructive and detailed comment. We agree that the previous Discussion section placed too much emphasis on practical recommendations and did not sufficiently develop the analytical and theoretical meaning of the findings. In the revised manuscript, we retained the original discussion structure based on the socio-ecological framework, but substantially strengthened the interpretation of the findings. Specifically, we clarified the mechanisms through which physical and psychological distress, relational burden and environmental barriers contribute to social isolation; expanded the comparison with existing literature; reduced overly prescriptive recommendation-style statements; added discussion of study limitations, context and transferability; and emphasized the theoretical contribution of understanding social isolation as a multi-level process involving individual withdrawal, relational disconnection and environmental exclusion.

• Limited analytical depth: Discussion largely restates the results using theoretical language, with insufficient deeper interpretation or exploration of underlying mechanisms.

**Response：**Thank you for this comment. We revised the Discussion to move beyond restating the themes and to explain the underlying mechanisms. In the revised version, we clarified that social isolation among older adults with T2DM is not only caused by reduced social contact, but develops through the interaction of illness-related vulnerability, changed interpersonal relationships and environmental exclusion. We also explained how physical decline and fear reduce social confidence, how overprotective family care and disease-centred relationships reduce autonomy and meaningful connection, and how inaccessible services, public spaces and stigma restrict social participation.

We strengthened the interpretive statement as follows: “The findings indicate that social isolation in this population is not merely a state of living alone or having fewer social contacts, but a cumulative process shaped by illness-related vulnerability, changes in interpersonal relationships and environmental exclusion. These levels interacted with each other: physical decline and fear of complications weakened participants’ confidence in social participation; family overprotection and disease-centred relationships reduced autonomy and narrowed social networks; and unsuitable community services, inaccessible public spaces and disease-related stigma further restricted opportunities for participation.”

• Weak engagement with literature: Existing studies are cited mainly for support, with little critical comparison or positioning of findings within broader research.

**Response：**We agree with this comment. In the revised Discussion, we did not simply use previous studies as supporting citations. Instead, we compared our qualitative findings with existing quantitative evidence and clarified how the present study extends previous research. For example, previous studies have reported associations between social isolation and poorer diabetes-related outcomes, whereas our study further explains how social isolation may be produced in daily life through role loss, fear of becoming a burden and weakened social identity.

“Previous studies have shown that social isolation is associated with poorer health outcomes among people with diabetes, including increased risk of microvascular complications and poorer glycaemic control. However, the present qualitative findings further explain how social isolation may be formed in daily life. For some older adults, withdrawal from social activities was not caused only by physical limitations, but also by fear of becoming a burden, reduced confidence and the feeling that their previous social identity had been weakened.”

• Overemphasis on recommendations: A substantial portion focuses on intervention strategies which are often overly prescriptive and not fully grounded in the data.

**Response：**We reduced overly prescriptive and policy-like recommendations and repositioned practical implications as a concise paragraph near the end of the Discussion. The revised Discussion now focuses more on interpretation and conceptual meaning, while the practice implications are presented as broad directions rather than detailed intervention protocols.

We revised the recommendations into a shorter implication statement:

“Therefore, interventions should not focus only on encouraging patients to participate in activities. Healthcare professionals should also assess patients’ emotional loneliness, perceived autonomy and social participation needs. Families should be guided to provide autonomy-supportive care rather than excessive protection. Communities should develop age-friendly and diabetes-sensitive services, including low-intensity activities, accessible registration methods and safe public spaces.”

• Lack of methodological reflection: There is no discussion of study limitations, context or transferability, leading to potential overgeneralization of findings.

**Response：**We added a limitations paragraph to acknowledge the single-site design, small qualitative sample and lack of longitudinal follow-up. We also revised the wording from “generalizability” to “transferability,” which is more appropriate for qualitative research. This revision clarifies the study context and avoids overgeneralizing the findings.

• Imbalance and structure issues: The section is uneven, with minimal interpretive discussion and extensive policy-like recommendations, reducing academic rigor.

**Response：**We adjusted the Discussion so that the main body focuses on interpretation rather than recommendations. The revised Discussion now emphasizes mechanisms such as loss of social identity, emotional isolation despite physical care, ambivalent family support, disease-centred social relationships, digital exclusion, inaccessible public spaces and stigma.

• Underutilization of qualitative insights: The richness of the data is not fully translated into higher-level conceptual or theoretical contributions.

**Response：**In the revised Discussion, we added a higher-level conceptual interpretation of the findings. Rather than treating social isolation as a simple outcome of disease burden, we now frame it as a process produced through the interaction of individual withdrawal, relational disconnection and environmental exclusion. This interpretation highlights the contribution of the qualitative data and strengthens the theoretical connection with the socio-ecological framework.

“Overall, this study contributes to the understanding of social isolation among older adults with T2DM by showing that it is produced through the interaction of individual withdrawal, relational disconnection and environmental exclusion. This interpretation moves beyond a simple risk-factor model and highlights the multi-level nature of social isolation.”

**Detailed responses to Reviewer 2**

**Reviewer #2:** 1. The study presents limited results of insufficient qualitative research with only 14 participants in the entire study.

**Response:**

Thank you for this important comment. We understand the reviewer’s concern regarding the sample size. In qualitative research, sample size is usually determined by information richness and data saturation rather than by statistical power calculation. In the revised manuscript, we clarified that purposive sampling was used to recruit participants who could provide rich information about social isolation among older adults with T2DM, and that maximum variation was considered in gender, age, marital status, educational level, occupational background and duration of T2DM. We also added a detailed description of information saturation.

We revised the Participant selection and recruitment section by adding an explanation of how data saturation was assessed:

“The sample size was determined by information saturation rather than by statistical calculation. Data collection and preliminary analysis were conducted concurrently. After each interview, the research team reviewed the transcript and field notes to determine whether new information, codes or categories emerged. After the twelfth interview, the main categories had become stable; two additional interviews were conducted to confirm saturation. As no new themes emerged from these additional interviews, data collection was stopped after 14 interviews.”

2. The participants characteristics are inadequately presented. There is need to present the mean age / standard deviation or median age and interquartile range.

**Response:**

Thank you for this helpful suggestion. We have revised Table 1 to present participant characteristics more clearly. Specifically, we changed the table title to “Demographic and disease-related characteristics of participants (n = 14)”, added the unit “years” to age, and clarified that the disease duration refers to the duration of type 2 diabetes mellitus by revising “Duration of illness (years)” to “Duration of T2DM (years)”. In addition, we added descriptive statistics for age and duration of T2DM before Table 1, including mean, standard deviation, median, interquartile range and range.

**We added the following text before Table 1:**

“The 14 participants ranged in age from 63 to 77 years, with a mean age of 71.00 ± 3.94 years and a median age of 71.00 years (interquartile range: 69.00–73.00 years). The duration of T2DM ranged from 6 to 18 years, with a mean duration of 11.71 ± 3.65 years and a median duration of 11.50 years (interquartile range: 8.50–14.75 years).”

**We also revised Table 1 to make the units clearer.**

3. The result column indicating duration of illness is unclear. What illness was considered?

**Response:**

Thank you for pointing this out. We agree that the term “duration of illness” was unclear. In the revised manuscript, we clarified that this variable refers to the duration since diagnosis of type 2 diabetes mellitus.

4. A single session of in-dept interview is inadequate to reach remarkable conclusions.

**Response:**

Thank you for this comment. We understand the reviewer’s concern. In this descriptive qualitative study, each participant completed one face-to-face semi-structured in-depth interview lasting approximately 45 minutes. During the interviews, the researcher used open-ended questions and probing questions to encourage detailed narration, recorded non-verbal information in field notes, and wrote reflective notes after each interview. Data collection and preliminary analysis were conducted concurrently until information saturation was reached.

We also revised the manuscript to avoid overly broad or definitive claims. The conclusions were softened to reflect the exploratory nature of the study.

**First, we expanded the Data collection section by clarifying the interview process:**

“Each interview lasted approximately 45 minutes. With participants’ permission, all interviews were audio-recorded. During the interviews, the researcher followed the interview guide flexibly, encouraged participants to describe their experiences in their own words, avoided leading questions and recorded non-verbal information such as emotional changes, pauses, facial expressions and gestures in field notes. After each interview, the researcher wrote reflective notes to record initial impressions, contextual information and potential influence of the researcher’s assumptions on data interpretation.”

**Second, we added this limitation in the Discussion section:**

“Because the study was based on one-time interviews, it could not capture how experiences of social isolation change over time with disease progression, family changes or community support. Future studies could use repeated interviews or longitudinal qualitative designs to further examine the dynamic process of social isolation among older adults with T2DM.”

5. Conclusions made are not valid because results are insufficiently presented from inadequate data.

**Response:**

Thank you for this critical comment. We revised the Results and Conclusion sections to ensure that all conclusions are closely grounded in the qualitative data. We avoided overgeneralized statements and presented the findings as exploratory qualitative insights rather than broadly generalizable conclusions. We also clarified that the three themes were derived from participants’ narratives and reflected the study context.

We revised the Conclusion section as follows:

“This study found that social isolation among older adults with type 2 diabetes is shaped by physical and psychological distress, relational burden and environmental barriers. The findings suggest that social isolation is not merely a lack of social contact, but a multi-level experience involving individual vulnerability, weakened interpersonal connections and restricted opportunities for social participation. Healthcare professionals should pay attention to patients’ emotional loneliness, autonomy and social participation needs, while families and communities should provide more supportive, inclusive and age-friendly environments. Future multi-centre and longitudinal studies are needed to further explore the dynamic mechanisms of social isolation and develop targeted intervention strategies for this population.”

6. The research methods and design have not referenced to justify the use of 14 participants for only a single session. This compromises the integrity of the whole study.

**Response:**

Thank you for this valuable suggestion. We revised the Methods section to better justify the descriptive qualitative design, purposive sampling strategy and sample size. We clarified that the purpose of this study was to obtain an in-depth understanding of participants’ experiences rather than to achieve statistical representativeness. We also added methodological references to support the use of information saturation as the basis for determining sample size in qualitative interview studies.

We added the following explanation in the Study design and Participant selection and recruitment sections:

“This study adopted a descriptive qualitative design to explore the experiences of social isolation among older adults with T2DM. A descriptive qualitative approach was considered appropriate because the purpose of the study was not to test a predetermined theory, but to obtain a direct and detailed understanding of how older patients perceive, experience and respond to social isolation in their everyday lives.”

and:

“In qualitative research, sample size is guided by the richness of information and information saturation rather than by statistical power calculation. Therefore, data collection and preliminary analysis were conducted concurrently, and recruitment continued until no substantially new codes or themes emerged from the interviews.”

We also added methodological references, such as Sandelowski, Vasileiou et al., and Hennink and Kaiser, to support the sample size and saturation justification.

7. The study requires major revisions and may only be accepted when additional information on the methods, the number of sessions of the in-dept interview is more than once.

**Response:**

Thank you for this recommendation. We have substantially revised the Methods section by adding more details on study design, purposive sampling, recruitment, interview procedures, interviewer training, reflexivity, data analysis, data saturation and quality assurance. Regarding the number of interview sessions, each participant completed one in-depth semi-structured interview. We acknowledge that repeated interviews may provide richer longitudinal data; therefore, we explicitly added this as a limitation and suggested repeated interviews or longitudinal qualitative designs in future research.

**We added or expanded the following parts in the revised manuscript:**

Study design: clarified the descriptive qualitative design and its rationale.

Participant selection and recruitment: added purposive sampling, maximum variation sampling and data saturation.

Data collection: added interview duration, interviewer training, audio recording, field notes and reflective notes.

Researcher reflexivity: added discussion of how researchers’ professional background might influence data collection and interpretation.

Data analysis: clarified qualitative content analysis, two-researcher coding, theme development, disagreement resolution and audit trail.

Limitations: added that one-time interviews may not capture dynamic changes over time.

**The limitation was revised as follows:**

“Although purposive sampling was used and data collection was guided by information saturation, the sample size was relatively small. In addition, because the study was based on one-time interviews, it could not capture how experiences of social isolation change over time. Future studies could use multi-centre sampling, repeated interviews, longitudinal qualitative designs or mixed-method approaches to further examine the dynamic mechanisms of social isolation among older adults with T2DM.”
