# Supplementary material for: Experiences of social isolation among older adults with type 2 diabetes mellitus: A qualitative study
Source: PLoS One. 2026 Aug 10;21(8):e0354450. doi: 10.1371/journal.pone.0354450 (PMC13456196; doi:10.1371/journal.pone.0354450)
Supplement: Supplementary file 10004 [file pone.0354450.s002.pdf]

# PLOS One

# Qualitative Study on Social Isolation Experience of Elderly Patients with Type 2 Diabetes Mellitus

--Manuscript Draft--

| | |
|---|---|
| **Manuscript Number:** | PONE-D-26-08610 |
| **Article Type:** | Research Article |
| **Full Title:** | Qualitative Study on Social Isolation Experience of Elderly Patients with Type 2 Diabetes Mellitus |
| **Short Title:** | Qualitative Study on Social Isolation Experience |
| **Corresponding Author:** | shanshan Hu<br>China Three Gorges University<br>宜昌市, 湖北省 CHINA |
| **Keywords:** | Key words: elderly; Type 2 Diabetes Mellitus; social isolation; experience; Qualitative research |
| **Abstract:** | Abstract: Objective: To explore the social isolation experience of elderly patients with type 2 diabetes mellitus (T2DM) and to provide a reference for nursing intervention. Methods: Fourteen elderly patients with type 2 diabetes mellitus were interviewed face-to-face using a semi-structured approach based on purposive sampling, and relevant data were collected. The data were analyzed by content analysis. Results: Three themes were summarized: physical and mental dilemma (reduced self-worth, anxiety and fear of the future, loneliness and emotional isolation), relational load (protective burden of family, alienation of companions and scene transition, supportive absence of doctor-patient communication), and environmental barriers (situational absence of community services, lack of friendliness in public spaces, disease stigma and social cognitive bias). Conclusion: The social isolation of elderly patients with type 2 diabetes mellitus has characteristic manifestations and is caused by many factors. It is suggested that medical staff should dynamically evaluate the state of patients, accurately identify the phenomenon of social isolation, formulate targeted intervention plans, mobilize an all-round social support system, and promote patients' active integration into society and return to normal life as soon as possible according to the actual situation and individual factors of patients. |
| **Order of Authors:** | shanshan Hu |
| | chaxiang Li |
| **Additional Information:** | |
| **Question** | **Response** |
| **Financial Disclosure**<br><br>Enter a financial disclosure statement that describes the sources of funding for the work included in this submission. Review the submission guidelines for detailed requirements. View published research articles from *PLOS ONE* for specific examples.<br><br>This statement is required for submission and **will appear in the published article** if the submission is accepted. Please make sure it is accurate. | The author(s) received no specific funding for this work. |

Powered by Editorial Manager® and ProduXion Manager® from Aries Systems Corporation

| | |
|---|---|
| **Funded studies**<br>Enter a statement with the following details:<br>• Initials of the authors who received each award<br>• Grant numbers awarded to each author<br>• The full name of each funder<br>• URL of each funder website<br>• Did the sponsors or funders play any role in the study design, data collection and analysis, decision to publish, or preparation of the manuscript?<br><br>Did you receive funding for this work? | |
| **Competing Interests**<br><br>Use the instructions below to enter a competing interest statement for this submission. On behalf of all authors, disclose any competing interests that could be perceived to bias this work—acknowledging all financial support and any other relevant financial or non-financial competing interests.<br><br>This statement is required for submission and **will appear in the published article** if the submission is accepted. Please make sure it is accurate and that any funding sources listed in your Funding Information later in the submission form are also declared in your Financial Disclosure statement.<br><br>View published research articles from *PLOS ONE* for specific examples. | The authors have declared that no competing interests exist. |

*Powered by Editorial Manager® and ProduXion Manager® from Aries Systems Corporation*

| | |
|---|---|
| **NO authors have competing interests**<br><br>Enter: *The authors have declared that no competing interests exist.*<br><br>**Authors with competing interests**<br><br>Enter competing interest details beginning with this statement:<br><br>*I have read the journal's policy and the authors of this manuscript have the following competing interests: [insert competing interests here]*<br><br>* typeset | |
| **Ethics Statement**<br><br>Enter an ethics statement for this submission. This statement is required if the study involved:<br><br>• Human participants<br>• Human specimens or tissue<br>• Vertebrate animals or cephalopods<br>• Vertebrate embryos or tissues<br>• Field research<br><br>Write "N/A" if the submission does not require an ethics statement.<br><br>General guidance is provided below. Consult the submission guidelines for detailed instructions. **Make sure that all information entered here is included in the Methods section of the manuscript.** | All subjects voluntarily participated in this study and signed an informed consent form. This study has been approved by the Ethics Committee of Yichang Central People's Hospital (Approval No.: 2025-304-01). |

| | |
|---|---|
| **Format for specific study types**<br><br>**Human Subject Research (involving human participants and/or tissue)**<br>• Give the name of the institutional review board or ethics committee that approved the study<br>• Include the approval number and/or a statement indicating approval of this research<br>• Indicate the form of consent obtained (written/oral) or the reason that consent was not obtained (e.g. the data were analyzed anonymously)<br><br>**Animal Research (involving vertebrate animals, embryos or tissues)**<br>• Provide the name of the Institutional Animal Care and Use Committee (IACUC) or other relevant ethics board that reviewed the study protocol, and indicate whether they approved this research or granted a formal waiver of ethical approval<br>• Include an approval number if one was obtained<br>• If the study involved *non-human primates*, add *additional details* about animal welfare and steps taken to ameliorate suffering<br>• If anesthesia, euthanasia, or any kind of animal sacrifice is part of the study, include briefly which substances and/or methods were applied<br><br>**Field Research**<br><br>Include the following details if this study involves the collection of plant, animal, or other materials from a natural setting:<br>• Field permit number<br>• Name of the institution or relevant body that granted permission | |
| **Data Availability**<br><br>Authors are required to make all data underlying the findings described fully available, without restriction, and from the time of publication. PLOS allows rare exceptions to address legal and ethical concerns. See the PLOS Data Policy and FAQ for detailed information. | Yes - all data are fully available without restriction |

| | |
|---|---|
| A Data Availability Statement describing where the data can be found is required at submission. Your answers to this question constitute the Data Availability Statement and **will be published in the article**, if accepted.<br><br>**Important:** Stating 'data available on request from the author' is not sufficient. If your data are only available upon request, select 'No' for the first question and explain your exceptional situation in the text box.<br><br>Do the authors confirm that all data underlying the findings described in their manuscript are fully available without restriction? | |
| **Describe where the data may be found in full sentences. If you are copying our sample text, replace any instances of XXX with the appropriate details.**<br><br>• If the data are **held or will be held in a public repository**, include URLs, accession numbers or DOIs. If this information will only be available after acceptance, indicate this by ticking the box below. For example: *All XXX files are available from the XXX database (accession number(s) XXX, XXX.).*<br>• If the data are all contained **within the manuscript and/or Supporting Information files**, enter the following: *All relevant data are within the manuscript and its Supporting Information files.*<br>• If neither of these applies but you are able to provide **details of access elsewhere**, with or without limitations, please do so. For example:<br><br>*Data cannot be shared publicly because of [XXX]. Data are available from the XXX Institutional Data Access / Ethics Committee (contact via XXX) for researchers who meet the criteria for access to confidential data.*<br><br>*The data underlying the results presented in the study are available from (include the name of the third party* | All relevant data are within the manuscript and its Supporting Information files. |

| | |
|---|---|
| *and contact information or URL).*<br>• This text is appropriate if the data are owned by a third party and authors do not have permission to share the data.<br><br>* typeset | |
| Additional data availability information: | Tick here if the URLs/accession numbers/DOIs will be available only after acceptance of the manuscript for publication so that we can ensure their inclusion before publication. |

*Powered by Editorial Manager® and ProduXion Manager® from Aries Systems Corporation*

Manuscript *手稿
Click here to access/download;Manuscript;renamed_ddd06.docx

# Title Page

**Manuscript Title:** Qualitative Study on Social Isolation Experience of Elderly Patients with Type 2 Diabetes Mellitus

**Authors:** Hu Shanshan[1,2], Li Chaxiang[1]

**Affiliations:**

1.First Clinical Medical College of Three Gorges University / Yichang Central People's Hospital, Yichang 443000, Hubei, China

2.College of Medicine and Health Sciences, Three Gorges University, Yichang 443000, Hubei, China

**First Author:**

Hu Shanshan

E-mail: LW3H2017@163.com

Phone: +86-18315861660

Address: No. 8 Daxue Road, Yichang City, Hubei Province, China

**Corresponding Author:**

Li Chaxiang

E-mail: 45595280@qq.com

**Acknowledgements:** This research received no specific grant from any funding agency in the public, commercial, or not-for-profit sectors.

**Competing Interests Statement:** The authors declare that there are no competing financial interests or personal relationships that could have appeared to influence the work reported in this paper.

**Author Contributions:**

Hu Shanshan: Overall study conception, methodology design, investigation and analysis, and manuscript draft writing.

Li Chaxiang: Research supervision, project administration, resource coordination, and final manuscript review and revision.

# Qualitative Study on Social Isolation Experience of Elderly Patients with Type 2 Diabetes Mellitus

**Abstract: Objective:** To explore the social isolation experience of elderly patients with type 2 diabetes mellitus (T2DM) and to provide a reference for nursing intervention. **Methods:** Fourteen elderly patients with type 2 diabetes mellitus were interviewed face-to-face using a semi-structured approach based on purposive sampling, and relevant data were collected. The data were analyzed by content analysis. **Results:** Three themes were summarized: physical and mental dilemma (reduced self-worth, anxiety and fear of the future, loneliness and emotional isolation), relational load (protective burden of family, alienation of companions and scene transition, supportive absence of doctor-patient communication), and environmental barriers (situational absence of community services, lack of friendliness in public spaces, disease stigma and social cognitive bias). **Conclusion:** The social isolation of elderly patients with type 2 diabetes mellitus has characteristic manifestations and is caused by many factors. It is suggested that medical staff should dynamically evaluate the state of patients, accurately identify the phenomenon of social isolation, formulate targeted intervention plans, mobilize an all-round social support system, and promote patients' active integration into society and return to normal life as soon as possible according to the actual situation and individual factors of patients.

**Key words:** elderly; type 2 diabetes mellitus; social isolation; experience; qualitative research

Type 2 Diabetes Mellitus (T2DM) [1]arises from dysfunction of pancreatic β-cells and target organ resistance to insulin, resulting in relative insulin deficiency. The Clinical Guidelines for the Prevention and Treatment of Type 2 Diabetes in the Elderly in China (2022 Edition) [2]report that 30% of individuals aged ≥60 years in China have diabetes, with type 2 diabetes accounting for over 95% of these cases. Compared to 1990, the overall mortality rate from T2DM among Chinese elderly individuals in 2021 increased by 197.98%, with female mortality rising by 158.61% and male mortality by 257.93% [3]. This demonstrates the disease's characteristics of prolonged duration, high incidence, and high mortality. As no curative medication currently exists, patients typically require lifelong medication or insulin injections alongside sustained self-management. Some develop complications affecting renal, cardiovascular, cerebrovascular, or peripheral vascular systems, leading to psychological distress such as anxiety and depression among elderly T2DM patients, which in turn triggers social disengagement. Social isolation [4]denotes a phenomenon where individuals, for various reasons, experience a psychological and behavioural self-imposed distancing and seclusion from social interactions. Social isolation exerts adverse effects on disease progression in elderly type 2 diabetes patients. Research indicates an independent association between social isolation and the risk of developing diabetic microvascular complications (DMCs). Notably, this association rivals that of other lifestyle factors (such as smoking, blood pressure, and physical activity). Collectively, these findings suggest social isolation constitutes a significant and potentially modifiable risk factor for DMCs in individuals with type 2 diabetes [5]. Furthermore, another study [6] revealed significantly higher blood glucose and glycated hemoglobin levels in the high social isolation group compared to the low isolation group, indicating that greater social isolation correlates with elevated fasting blood glucose and glycated hemoglobin levels. However, to date, research on social isolation among elderly patients

with type 2 diabetes in China remains limited overall, predominantly comprising quantitative studies [7-8]. In light of this, the present study employs a descriptive qualitative research approach. Through in-depth interviews with this specific cohort of elderly individuals with type 2 diabetes, it explores their authentic experiences of social isolation. This aims to provide a reference basis for healthcare professionals in formulating social isolation intervention programmes.

## 1 Subjects and Methods

### 1.1 Study Population

From November 10, 2025 to December 31, 2025, elderly patients with type 2 diabetes mellitus attending the Endocrinology Department of a tertiary-level Class A hospital in Yichang City were selected via purposive sampling for semi-structured in-depth interviews. Inclusion Criteria: □ Meeting diagnostic criteria outlined in the Chinese Diabetes Society's 2017 Guidelines for the Prevention and Treatment of Type 2 Diabetes (2017 Edition). □ Age ≥ 60 years. □ Clear consciousness with capacity to participate in the study. □ Informed consent obtained. Exclusion criteria: History of psychiatric disorders or cognitive impairment; hearing or language communication barriers; refusal to participate. Sample size was determined by information saturation, with 14 participants ultimately included for analysis. The 14 elderly type 2 diabetes patients interviewed were designated N1-N14 to replace their real names. Basic participant information is presented in Table 1. All subjects voluntarily participated and signed informed consent forms.

**Table 1: Basic Information of Respondents (n=14)**

| Number | Gender | Age | Marital status | Educational attainment | Occupational background | Duration of illness |
|---|---|---|---|---|---|---|
| N1 | Male | 73 | Married | Technical secondary school | Workers | 12 |
| N2 | Female | 76 | Married | Primary school | Self-employed individuals | 8 |
| N3 | Male | 71 | Widowed | Primary school | Farmers | 15 |
| N4 | Male | 72 | Married | Junior secondary school | Workers | 11 |
| N5 | Female | 69 | Married | College | Teachers | 6 |
| N6 | Male | 71 | Married | Junior secondary school | Workers | 7 |
| N7 | Male | 76 | Widowed | Primary school | Farmers | 8 |
| N8 | Male | 73 | Widowed | College | Civil servants | 10 |
| N9 | Female | 77 | Married | Junior secondary school | Workers | 11 |
| N10 | Male | 68 | Married | Technical | Farmers | 15 |

| Number | Gender | Age | Marital status | Educational attainment | Occupational background | Duration of illness |
|---|---|---|---|---|---|---|
| | | | | secondary school | | |
| N11 | Female | 66 | Married | Primary school | Workers | 13 |
| N12 | Female | 70 | Widowed | Primary school | Self-employed individuals | 14 |
| N13 | Male | 69 | Married | Junior secondary school | Workers | 16 |
| N14 | Female | 63 | Married | Junior secondary school | Workers | 18 |

### 1.2 Research Methodology

#### 1.2.1 Development of Interview Guide

Employing a descriptive qualitative research approach, the interview guide was designed within the framework of social ecological theory following a review of relevant literature and discussions within the research team. This theory emphasises that individual behaviour and experiences are shaped by the interplay of multi-level systems: micro (individual), meso (interpersonal), and macro (environmental and societal). To operationalise the theory, the research team systematically translated its three levels into interview questions. Following preliminary interviews with three participants, the outline was revised. The final formal interview questions are as follows: □ What physical and psychological changes have you experienced since becoming ill, and how have these affected your social interactions? □ How has the attitude of family, friends, etc., towards you changed compared to before your illness? □ When attending medical appointments, how attentive were healthcare professionals to your psychological state? □ Do you typically participate in community activities? What factors influence your participation? □ Do you feel others' perceptions of this illness affect your willingness to engage in activities? □ What forms of support would you like to receive?

#### 1.2.2 Data Collection and Quality Control Methods

Data were gathered through face-to-face semi-structured interviews. Prior to interviews, researchers undertook systematic training in qualitative research methodologies. Trusting relationships were established with participants, who were fully informed of the study's content and objectives. Following informed consent and the signing of consent forms, interviews were conducted in quiet, undisturbed demonstration rooms. Throughout the interviews, a voice recorder captured the entire conversation. Researchers followed the interview guide, listened attentively to participants' narratives, and carefully observed and documented non-verbal cues such as facial expressions, gestures, and tone of voice. Interruptions or leading language/actions were avoided, with each session lasting no longer than 30 minutes. Following each interview, corresponding materials were catalogued, securely stored by designated personnel, and interview notes were compiled.

### 1.2.3 Data Analysis Methodology

Following the interviews, two postgraduate students transcribed the recordings verbatim into text within 24 hours, integrating the interviewees' facial expressions and body language to form the final textual data. The text was imported into Nvivo 12.0 software and analysed using content analysis methodology. Through careful and repeated reading of the interview materials, key viewpoints were marked and open coding applied after gaining an overall grasp of the text content. Subsequently, similar or related codes were categorised into themes and sub-themes, undergoing iterative adjustments through decomposition or the addition of new elements to ultimately establish core themes.

## 2 Findings

### 2.1 Physical and Psychological Distress

### 2.1.1 Reduced Sense of Self-Worth

Elderly patients with type 2 diabetes experience feelings of uselessness due to disease-related physical decline, increased dependence on others, and withdrawal from social or family roles, which impedes normal social interaction. N1: In my younger days, I was a foreman in the workshop, supervising over twenty people. When machinery malfunctioned, I could pinpoint the fault with my eyes closed. Now I'm no good for anything. N2: Neighbours used to ask me to tailor clothes for them. Now my hands sometimes go numb, I can't hold scissors steady, and my eyesight's gone terribly blurry. They've gradually stopped coming to me. N6: I used to be the tenor in our community seniors' choir. Now I just watch from afar. It's not that I don't want to go---it's that I feel like a burden when I do. N8: Before retirement, I was a minor manager overseeing dozens at work. I was decisive and efficient in everything I did. But now... (he shook his head) Now I feel like an old file forgotten in a warehouse corner, never to be looked at again.

### 2.1.2 Anxiety and Fear of the Future

Some interviewees expressed persistent anxiety about their condition's progression and future livelihood. This anxiety stemmed not only from physical fears of complications but extended to long-term concerns about family relationships, financial burdens, and personal dignity, becoming an invisible pressure permeating daily life. N1: Today I fear high blood sugar, tomorrow I fear complications. Living like this... (Voice trails off) ...truly devoid of flavour. N10: I live in constant dread now, fearing sudden spikes in blood sugar or complications catching up with me. When my legs go numb, I wonder if diabetic foot is approaching. This anxiety never ceases---it even haunts my sleep. N12: My children have their own families; I don't want to be a burden. Yet the thought of needing care if my condition worsens fills me with profound distress. N13: Monthly medication and test fees are a significant expense. Though manageable now, what if my condition worsens and costs escalate? I genuinely fear I won't be able to afford treatment later.

### 2.1.3 Loneliness and Emotional Isolation

Some interviewees receive regular visits or daily care, yet their disease experiences, psychological burdens, and emotional needs often lack genuine listening, understanding, or empathy. They sink into a silence where no one can hear their story. N8: This loneliness isn't about whether people are physically present. My son and daughter-in-law bring my grandson to visit at weekends, and the house buzzes with activity. But once they leave, that silence carries an echo (his voice grows hushed). I have so much I want to say, but nowhere to say it. N7: My children are all busy. If they

manage to visit once a week, that's good enough. Most of the time, I'm alone in this empty house. N3: I know the children are busy with work. They hired a carer for me who comes twice a day to prepare meals. That carer, well, she just makes sure I don't fall or go hungry. But I want someone to talk to.

### 2.2 Relational Burden

#### 2.2.1 The Protective Burden of Family

Family members, driven by health anxieties and risk avoidance, often impose excessive protection and control. This management, cloaked in care, frequently manifests as strict restrictions on the patient's daily activities, financial autonomy, and social participation. Though well-intentioned, it objectively compresses the patient's space for independent decision-making, potentially intensifying the inherent conflict between dependence and autonomy. N3: My daughter took my ID card and pension card, saying she fears I'll buy unhealthy food or be tricked into buying health supplements. If I go out walking for more than half an hour, she calls to check up on me. N10: Every time I want to go downstairs to the chess room to watch people play, my wife sighs and says, 'With your high blood sugar, why don't you just stay home? What if you faint outside?' 'You may not care for yourself, but think how worried I and the children are!' N11: The community organised a senior citizens' trip, and I was keen to go. My wife declined on my behalf, then told me, 'You couldn't manage those group meals, and the journey would be exhausting. I've turned it down for you.' N14: I'm nothing but a figurehead now. My son orders my meals online, my daughter-in-law divides my pills into compartments, and they even schedule when I go downstairs for my daily sun exposure. This love feels like a heavy burden to me.

#### 2.2.2 The Drift of Companions and Shifting Contexts

Some patients often withdraw, either actively or passively, from their original social circles built on shared interests or life experiences. New social connections frequently form around exchanging disease information and experiences, resulting in a narrowed scope of social participation and a forced shift in life's focus. N8: I used to have a few old mates who'd meet up once or twice a week. Ever since I was diagnosed with diabetes, I can't drink alcohol or eat greasy food, so they don't invite me out as much. Now I often attend diabetes-related lectures in my spare time to learn more about it. N14: Before falling ill, I had a regular group of dance partners. After dancing, we'd shop for groceries together or stroll in the park. Now my stamina can't keep up, so I gradually withdrew. Later, I joined a diabetes WeChat group where members constantly discuss which medications work best or which pumpkin varieties cause slower blood sugar spikes. N12: My best friend and I used to chat on the phone for hours, discussing all manner of domestic matters. Now, whenever she calls, it's straight to 'How's your blood sugar?' or 'Is your foot numb?' After these exchanges, there's an awkward silence before we politely hang up.

#### 2.2.3 Supportive Gap in Doctor-Patient Communication

Healthcare professionals' highly technical and task-oriented communication may overlook patients' psychological needs, thereby intensifying feelings of helplessness and loneliness. N6: Each follow-up appointment feels like moving through an assembly line. The nurse takes my blood pressure and measures my blood sugar, while the doctor glances at the figures on the screen and asks, 'Have you been taking your medication on time? What have your blood sugar levels been lately?' N12: The doctor always advises me to exercise moderately, but what constitutes "moderate"? Where should I exercise? He never elaborates. N13: Specialist appointments at large

hospitals are so difficult to secure. After finally getting one, you find a room full of people waiting. Even if I wanted to mention I've been sleeping poorly lately, he'd likely cut me off, saying it's not directly related to blood sugar and directing me to complete these tests first. In that atmosphere, you feel discussing your emotions and loneliness is a waste of time.

### 2.3 Environmental Barriers

#### 2.3.1 Contextual Shortcomings in Community Services

Community-organised activities tend to be generic, short-term, and overly technical, failing to effectively support social participation for this group. They overlook their inner needs and hinder patients from building social connections. N7: The community keeps organising walking events and hill-climbing competitions, supposedly to encourage older people to get moving. But for us with unstable blood sugar and weak legs, that's not exercise -- it's a risk. N8: Last year on World Diabetes Day, the community did organise a lecture with a doctor. But it was all grand principles, and once finished, everyone dispersed without time for questions. It felt like ticking off a task. N12: Now all community notices and event sign-ups are through WeChat groups. My eyesight is failing, I'm not adept with smartphones, and my children aren't nearby. Last time I wanted to join a health exercise class, they required registration via a mini-programme chain. Unable to navigate it, I had to give up.

#### 2.3.2 Insufficient Accessibility in Public Spaces

Current spatial designs often fail to adequately consider the specific needs of elderly individuals with chronic conditions. This results in a significant reduction of social interaction opportunities they could otherwise enjoy, due to physical barriers and safety concerns. N4: I rarely take the bus now. The stops are too far apart, and sometimes I wait over ten minutes. Standing for so long makes my legs go weak and my heart race---I dread a sudden hypoglycaemic episode. N8: Parks look lovely, but the toilets are miles apart. With our condition, frequent urination means we can't hold it in. Every visit involves calculating how far we are from the loo, which ruins the enjoyment. N11: The stairs at the community centre are far too steep, and there's no lift. My knees aren't good, and when my blood sugar's unstable, going up and down makes me dizzy and light-headed. Went once and never want to go back.

#### 2.3.3 Disease Stigma and Social Cognitive Biases

Misunderstandings, wariness, and low tolerance from society compel patients to avoid embarrassment, blame, or becoming a burden. They proactively choose silence, concealment, and withdrawal in social settings, thereby actively or passively severing connections with others at the interpersonal level. This ultimately exacerbates their marginalisation and isolation within social relationships. N2: Sometimes when I bump into old neighbours who know I have diabetes, their first words are, 'Oh dear, did you eat too much sweet stuff before?' It's as if this illness is my own fault, a punishment for lacking self-discipline. N7: Once, sitting on a park bench, it was time for my insulin injection. As soon as I took out the needle, the person sitting beside me recoiled as if they'd seen something terrifying and quickly moved away. That look made me feel as though I were doing something shameful. N11: Our neighbourhood dance troupe was rehearsing, and I wanted to watch. The leader pulled me aside and said, "Sister Wang, are you sure you're up to it? Don't faint from standing too long -- we can't take responsibility for that." The implication was clear: I was a burden. I haven't been back since. N13: My diabetes causes blurred vision, so I process things slowly. Once at the supermarket checkout, I took a moment counting my change.

The person behind muttered impatiently, "If you're too old, stay home and stop holding things up." It cut deep. They didn't know my condition; to them, I was just a useless old fool. Such misunderstandings make me increasingly afraid of crowded places where quick reactions are needed.

## 3 Discussion

This study analyses the experiences of social isolation among elderly patients with type 2 diabetes from a socio-ecological systems perspective. This theory posits that individual behaviour and experiences are shaped by the interplay of multi-level environmental systems: the micro-system (individual physical and mental states), meso-system (family, peer groups, doctor-patient relationships), and macro-system (community environment, socio-cultural factors). The social isolation experienced in this study emerges from the combined effects of these three systems: physical and psychological distress reflects shifts in cognitive and emotional states within the microsystem; relational burdens reveal gaps in supportive relationships within the mesosystem; while environmental barriers correspond to exclusionary physical and social conditions within the macrosystem. These factors do not operate in isolation but mutually reinforce each other, collectively forming a vicious cycle of social isolation for patients. Future interventions should focus on three key areas:

### 3.1 Prioritising Psychological Support and Empowerment Interventions to Break Internalised Barriers

In this study, participants reduced social engagement due to heightened negative emotions such as diminished self-worth, anxiety, and fear. Targeted psychological support measures are urgently required, including individual or group counselling. In individual counselling, professional psychologists can establish one-to-one trust-based relationships with patients to explore deep-seated issues such as self-denial and the roots of anxiety. Through cognitive behavioural therapy and similar approaches, they can assist patients in identifying and altering negative thought patterns, reconstructing positive self-perceptions, and enhancing self-worth [9-10]. Group counselling fosters an atmosphere of mutual understanding and support, enabling patients to share experiences and feelings. This facilitates empathy and encouragement from peers, alleviating loneliness and anxiety [11-12]. Concurrently, empowerment interventions prove vital. Disease self-management training courses can instruct patients in scientifically monitoring blood glucose levels, adopting balanced diets, and engaging in appropriate exercise. This empowers individuals to gain control over their health through mastering disease management skills, thereby boosting self-confidence [13-15]. Furthermore, interest group activities tailored to elderly type 2 diabetes patients can be organised. By leveraging participants' interests and talents, these activities enable individuals to discover joy and a sense of accomplishment in life, facilitating their reintegration into social life [16].

### 3.2 Establishing Supportive Interpersonal Interactions to Reduce Relational Burden

This study reveals that social isolation among elderly type 2 diabetes patients is significantly influenced by shifts in core social relationships. The sense of being controlled and limited social connections stemming from relational burdens constitute the core predicaments driving their isolation. Urgent action is required to construct supportive interpersonal networks across three key dimensions: family, peer groups, and doctor-patient relationships. At the familial level, the family serves as the most fundamental support system for elderly patients with type 2 diabetes, with its

caregiving patterns directly influencing patients' psychological states and willingness to participate in society. The sense of being controlled due to overprotection mentioned by some patients in this study aligns with the findings of Tang Huan et al. [17]in their research on chronic obstructive pulmonary disease. That study indicated that excessive protection by caregivers reduces the roles undertaken by elderly family members and diminishes their willingness to engage socially. Perceived control over one's personal life serves as a crucial indicator for regulating psychological stress. Enhancing patients' sense of control over their lives fosters greater self-management initiative and positive coping abilities [18]. Therefore, caregivers should be guided to shift their cognitive approach, establishing a collaborative care model that encourages patient involvement in household health decisions. This approach not only ensures scientifically sound disease management but also reinforces the patient's agency by respecting their autonomy. At the peer support level, science education programmes for companions should be implemented to help them understand patient needs. Guidance should focus on providing timely, appropriate companionship and activity participation, avoiding excessive disease-centred attention to preserve patient autonomy and the pleasure of social interaction. Additionally, exploring novel interaction models should encourage a shift towards low-burden activities such as emotional exchange and shared interests. Communication tools should be utilised effectively to maintain connections, deepening friendships despite disease limitations. At the clinician-patient level, interactions urgently require transformation into supportive partnerships. Promote patient-centred communication training, encouraging healthcare professionals to employ empathy and active listening. Proactively inquire about patients' psychosocial status to create a safe environment for expression. Structurally integrate psychosocial assessments into clinical pathways using brief screening tools, ensuring comprehensive needs receive attention.

### 3.3 Breaking Down Environmental Barriers: Building Inclusive Physical and Social Environments

#### 3.3.1 Strengthening Community Services

This study reveals that community public services fail to align with the specific needs of elderly Type 2 diabetes patients, instead creating participation barriers that exacerbate their social isolation. The universal design of community activities overlooks the health limitations of chronic disease groups, contradicting the principles of inclusivity and functional support advocated for healthy ageing[19]. This inadvertently conveys exclusionary signals, reinforcing patients' self-marginalisation. N8's experience illustrates how lectures often become perfunctory, lacking sustained interaction and support to meet chronic disease management's long-term requirements. More critically, as exemplified by N12's predicament, when service access heavily relies on smartphones, it creates digital exclusion for older adults unfamiliar with digital technology -- an emerging risk factor exacerbating social isolation[20]. Breaking these environmental barriers hinges on establishing a collaborative mechanism linking needs assessment, design, and evaluation, transforming elderly diabetes patients from service recipients into design participants. For instance, co-designing safe, gentle walking routes and pacing incorporates health limitations into the programme from the outset. This not only ensures accessibility and safety but, through the process itself, conveys signals of inclusivity, rebuilding a sense of social belonging. Addressing the digital divide requires technology-enabled interventions. Communities may appoint digital care coordinators to provide one-to-one, non-judgmental guidance on device usage. This extends beyond skill transfer to reducing technological anxiety through regular engagement, transforming

digital access scenarios into opportunities for building supportive interpersonal relationships. Service sustainability hinges on transitioning from one-off activities to support networks, such as establishing diabetes peer support groups that integrate disease management techniques with social interaction during regular gatherings. This model creates safe spaces for experience sharing, fostering organic informal support networks that effectively counteract self-isolation arising from disease management [21].

### 3.3.2 Enhancing Age-Friendly Design in Public Spaces

Inadequate age-friendliness in public spaces significantly hinders social engagement among older adults with type 2 diabetes. Issues such as inadequate barrier-free design in public facilities, insufficient seating, and unsafe walking paths exacerbate older adults' fear of falling, directly reducing their willingness to engage in outdoor activities. This aligns with research by Liu, Lin, Feng et al. [22], which highlighted a significant negative correlation between social isolation and fear of falling among community-dwelling older adults with type 2 diabetes, suggesting environmental risks inhibit social behaviour by affecting perceived safety. Future efforts should optimise physical space design by installing non-slip surfaces, rest benches, and accessible ramps to reduce fall risks, thereby encouraging outdoor engagement. Research indicates[23] that enhancing public space safety can diminish loneliness and elevate well-being among older adults.

### 3.3.3 Addressing Sociocultural Biases

Disease stigma stems not only from knowledge gaps but also from deeply ingrained cultural attribution biases. For instance, the public often simplistically attributes type 2 diabetes to personal lifestyle choices, overlooking its complex physiological, genetic, and environmental factors. This moralising attribution labels patients as lacking self-control rather than recognising it as a medical condition requiring support. When such judgements are conveyed through verbal and non-verbal means, they constitute social disease stigma; patients perceive and internalise these negative evaluations, leading to stigmatisation. Research by Jiang Lu et al. [24]explicitly identifies this sense of shame as a core predictor of social isolation among elderly type 2 diabetes patients. The resulting behavioural withdrawal and social avoidance, as noted by Hu Tiantian et al.[25], severely undermines patients' social support systems and role participation. For older adults, whose social networks are inherently fragile, this can precipitate their disintegration, trapping individuals in a vicious cycle of isolation, psychological distress, and diminished disease management efficacy. Public education must therefore address the cognitive roots of stigma: firstly, by deconstructing erroneous attributions and scientifically communicating the multifaceted and complex aetiology of the disease; secondly, by reshaping perceptions of patients through media narratives and community role models that portray their everyday lives of proactive disease management. This fosters public understanding and empathy, fundamentally dismantling stigma.

## 4 Conclusion

Through qualitative interviews, this study reveals that social isolation among elderly patients with type 2 diabetes is primarily influenced by the interplay of three factors: physical and psychological distress, relational burdens, and environmental barriers. This indicates the issue exhibits multi-systemic characteristics, necessitating the development of a multi-level, coordinated intervention system. At the micro level, psychological empowerment and self-management support should be strengthened to enhance patients' confidence and willingness to participate in society. At the meso level, improving interactions within families, peer groups, and doctor-patient

relationships can alleviate relational burdens and foster supportive social networks. At the macro level, promoting community services, adapting public spaces for ageing-friendly use, and conducting public education initiatives can cultivate an inclusive social environment. This study also has certain limitations. Firstly, employing qualitative research methods with a small sample size drawn from a single hospital in one region means the generalisability of conclusions requires further validation. Secondly, the study did not dynamically track the evolution of patients' experiences of social isolation. Future research could explore the following avenues: first, conducting multi-centre, large-sample mixed-method studies; second, undertaking longitudinal tracking to investigate the dynamic changes and long-term impacts of social isolation; third, developing and testing targeted, multi-level social integration intervention programmes based on the findings of this study, thereby providing evidence-based guidance for clinical practice and policy formulation.

**References:**

[1] Clinical Guidelines for the Prevention and Treatment of Type 2 Diabetes in the Elderly in China (2022 Edition) [J]. Chinese Journal of Diabetes, 2022, 30(1): 2-51.

[2] CHATTERJEE S, KHUNTI K, DAVIES M J. Type 2 diabetes [J]. Lancet, 2017, 389(10085): 2239-2251.

[3] ZHAO Xiaoxiao, KE Lixin, XUN Yangqin, et al. Global and Chinese burden of disease survey and future trend projections for type 2 diabetes in older adults, 1990–2021 [J]. Chinese General Practice, 2025, 28(16): 2050-2058.

[4] LIANG YQ, LIN Y, HUANG LL, et al. Current status and influencing factors of social isolation among cervical cancer survivors[J]. Journal of Nursing Science, 2022, 37(16): 64-67.

[5] LIANG Y Y, HE Y, WANG J, et al. Social Isolation, Loneliness, and Risk of Microvascular Complications Among Individuals With Type 2 Diabetes Mellitus[J]. American Journal of Kidney Diseases, 2024, 84(5): 557-566.e1.

[6] LU Q, QU L, XIE C, et al. Relationship between social isolation and glycaemic control of people previously diagnosed with diabetes: secondary analysis from the CHARLS[J]. BMJ Open, 2024, 14(3): e076106.

[7] Zhang K H, Li W L, Wang Y X, et al. Mediating effects of psychological flexibility and exercise self-efficacy on frailty and social isolation in elderly patients with type 2 diabetes mellitus[J]. Preventive Medicine, 2025, 37(8): 783-788.

[8] Zhou Mengjiao, Li Meilin, Kong Liping. Factors influencing social isolation among elderly patients with type 2 diabetes mellitus [J]. Preventive Medicine, 2024, 36(4): 292-295.

[9] Wu Ying, Duan Hongwei, Chen Xuan. Meta-analysis of cognitive behavioural therapy in improving depression and blood glucose levels among diabetic patients[J]. Journal of Nursing Science, 2018, 33(3): 23-27.

[10] NG T K, WONG D F K. The efficacy of cognitive behavioural therapy for Chinese people: a meta-analysis[J]. Australian and New Zealand Journal of Psychiatry, 2018, 52(7): 620-637.

[11] Li Fei, Li Mei, Liu Yandi, et al. Application of group nostalgia therapy in diabetic patients with depression [J]. Journal of Changchun University of Chinese Medicine, 2019, 35(5): 964-967.

[12] Duan Tian, Wang Zhe, Yin Aidi. Effects of positive psychology group counselling on exercise phobia, post-traumatic growth and quality of life among coronary heart disease patients following percutaneous coronary intervention [J]. Chinese Journal of Health Psychology, 2025, 33(6): 865-870.

[13] Tong Rui, Miao Jia, Gao Jingjing. Nursing Effects of LEARNS Health Education Intervention on Type 2 Diabetes Patients and Its Impact on Quality of Life Scores [J]. China Medical Guide, 2024, 21(16): 68-71.
[14] Fang Yunyan, Qin Wen, Wang Xiaobei, et al. Evaluation of the Impact of MDT Combined with Comprehensive Health Education on Elderly Diabetes Patients Using Propensity Score Matching [J]. Chinese Journal of Geriatrics, 2023, 43(17): 4339-4342.
[15] Fan Ying, Li Xiuming, Zou Dongyin, et al. Effects of an Internet-Based Interactive Chronic Disease Management Model on Glycaemic Control in Type 2 Diabetes Patients [J]. Western Chinese Journal of Traditional Medicine, 2023, 36(1): 124-127.
[16] Ma Jin, Tian Wenfeng, Xu He, et al. Advances in the Application of Art Therapy in Mental Health Services for Community-dwelling Elderly [J]. Journal of Nursing Science, 2025, 40(21): 125-129.
[17] Tang Huan, Chen Guihua, Li Qin, et al. Analysis of Post-Discharge Exercise Behaviour Characteristics and Predictors Among Patients with Chronic Obstructive Pulmonary Disease [J]. Chinese Journal of Nursing, 2024, 59(17): 2084-2092.
[18] Ji Yongjuan, Song Shuli, Wang Zhigang, et al. Study on the Intervention Effect of a Family Empowerment Programme on the Level of social isolation in Patients with Permanent Intestinal Stoma [J]. Journal of Nurse Training, 2023, 38(18): 1689-1693.
[19] Wang Xinyuan. Research on the Current Status of Healthy Ageing and Nursing Needs Preferences among Community-dwelling Elderly in the Context of Integrated Medical and Nursing Care [D]. Zhengzhou University, 2023.
[20] Yang Qiaoling, Liu Hui, Duan Yajuan, et al. Chain Mediation Effects of Social Isolation and Depression on Digital Exclusion and Cognitive Function Among Rural Elderly [J]. Journal of Nursing Science, 2025, 40(15): 23-28.
[21] Chinese Guidelines for Diabetes Prevention and Treatment (2024 Edition) (Excerpt I) [J]. Chinese Journal of Practical Internal Medicine, 2025, 45(10): 864-874+892.
[22] Liu, L. F., Zhang, W., Ma, W. L., et al. Correlation Study of Social Isolation, Social Support and Fear of Falling in Elderly Type 2 Diabetes Patients [J]. Chinese Journal of Sanatorium Medicine, 2025, 34(8): 16-19.
[23] Wang Dewen, Ren Jie. On Constructing Age-Friendly Communities in the Context of Population Ageing [J]. Journal of Xiamen University (Philosophy and Social Sciences Edition), 2015(5): 125-135.
[24] Jiang Lu, Su Qiong, Zheng Hongling, et al. Analysis of the Current Status and Influencing Factors of Social Isolation Among Type 2 Diabetes Patients [J]. Chongqing Medicine, 2024, 53(7): 1115-1120.
[25] Hu Tiantian, Jin Yilin, Jiang Tao, et al. A Qualitative Study on the Experiences of social isolation Among People Living with HIV/AIDS [J]. Journal of Nurse Training, 2021, 36(24): 2294-2299.
