# Supplementary material for: Experiences of social isolation among older adults with type 2 diabetes mellitus: A qualitative study
Source: PLoS One. 2026 Aug 10;21(8):e0354450. doi: 10.1371/journal.pone.0354450 (PMC13456196; doi:10.1371/journal.pone.0354450)
Supplement: Supplementary file 6 [file pone.0354450.s001.doc]

**Interview Transcripts**

| N1 | 1. I used to supervise more than twenty workers and could identify machine faults with my eyes closed. Now I feel useless. 2. Today I fear high blood sugar; tomorrow I fear complications. Living like this… truly feels tasteless |
| --- | --- |
| N2 | 1. Neighbours used to ask me to tailor clothes for them. Now my hands go numb, my eyesight is blurred, and they have gradually stopped coming. 2. When neighbours know I have diabetes, they ask whether I used to eat too many sweets, as if the illness were my own fault. |
| N3 | 1. My children hired a caregiver who comes twice a day to prepare meals. She makes sure I don’t fall or go hungry, but what I really want is someone to talk to. 2. My daughter took away my ID card and pension card, and even calls if I walk outside for more than half an hour. |
| N4 | 1.I rarely take the bus now. The stops are far apart, and waiting too long makes my legs weak. I fear a sudden hypoglycaemic episode. |
| N5 | 1.The diabetes lecture was full of general principles. After it ended, everyone left, and there was no time for questions. It felt like ticking off a task. |
| N6 | 1. I used to sing tenor in our community seniors’ choir. Now I only watch from afar—I feel like a burden when I join them. 2. Each follow-up feels like an assembly line: the nurse checks my blood pressure and blood sugar, and the doctor only asks whether I have taken my medication. |
| N7 | 1. My children are all busy. If they can visit once a week, that is already good enough. Most of the time, I am alone in this empty house. 2. The community organizes walking and hill-climbing events, but for people like us with unstable blood sugar and weak legs, that is not exercise—it is a risk. 3. Once, when I took out my insulin needle in a park, the person beside me quickly moved away. That look made me feel ashamed. |
| N8 | 1. Before retirement, I managed dozens of workers and made decisions efficiently. Now I feel like an old file forgotten in a warehouse corner, never to be looked at again. 2. This loneliness isn’t about whether people are physically present. My son and daughter-in-law bring my grandson to visit on weekends, and the house becomes lively. But once they leave, the silence seems to echo. I have so much I want to say, but nowhere to say it. 3. I used to meet old friends once or twice a week. After being diagnosed with diabetes, I could no longer drink or eat greasy food, so they gradually invited me less often. |
| N9 | 1.Parks look nice, but the toilets are too far apart. Every visit means calculating how far I am from the toilet. |
| N10 | 1. I live with constant fear of blood sugar spikes and complications. When my legs go numb, I worry about diabetic foot, and the anxiety follows me even into sleep. 2. Whenever I want to watch people play chess downstairs, my wife tells me to stay home because of my high blood sugar. |
| N11 | 1. I wanted to join a senior citizens’ trip, but my wife declined on my behalf. 2. The stairs at the community centre are too steep, and there is no lift. When my blood sugar is unstable, climbing them makes me dizzy. 3. I wanted to watch the neighbourhood dance rehearsal, but the leader worried I might faint and said they could not take responsibility. I felt like a burden and never went back. |
| N12 | 1. My children have their own families; I don’t want to become a burden. But the thought of needing care if my condition worsens deeply distresses me. 2. My best friend and I used to talk for hours. Now she only asks about my blood sugar or whether my foot is numb, and then we fall into awkward silence. 3. The doctor tells me to exercise moderately, but never explains what ‘moderate’ means or where I should exercise. 4. Community notices and registrations are now all on WeChat. I wanted to join a health exercise class, but I could not use the mini-programme, so I had to give up. |
| N13 | 1. Monthly medication and test fees are already a significant expense. If my condition worsens and costs increase, I fear I may not be able to afford treatment. 2. Specialist appointments are hard to get, and the room is always crowded. Even if I want to mention poor sleep or loneliness, I feel there is no time or space to talk about it. 3. Because of blurred vision, I move slowly at the supermarket checkout. When someone complained that I was holding things up, I felt like a useless old fool. |
| N14 | 1. My son orders my meals, my daughter-in-law sorts my pills, and they even schedule when I go downstairs for sun exposure. This love feels heavy. 2. I used to have regular dance partners, but my stamina could no longer keep up. Later, I joined a diabetes WeChat group where people mostly discussed medications and blood sugar control. |

This file contains selected original interview excerpts from 14 older adults with type 2 diabetes mellitus (coded as **N1–N14**) who participated in this qualitative study. The excerpts cover participants’narratives regarding social isolation, illness experiences, interpersonal relationships, healthcare encounters, and restrictions in daily life. These raw data support the thematic analysis presented in the main text and are provided to enhance the credibility and transparency of our findings, as well as to offer readers contextual depth. All personal identifiers have been anonymised, and only core statements relevant to the research topic are retained.
