# Supplementary material for: Experiences of social isolation among older adults with type 2 diabetes mellitus: A qualitative study
Source: PLoS One. 2026 Aug 10;21(8):e0354450. doi: 10.1371/journal.pone.0354450 (PMC13456196; doi:10.1371/journal.pone.0354450)
Supplement: Supplementary file 4 [file pone.0354450.s003.docx]

Qualitative Study on Social Isolation Experience of Elderly Patients with Type 2 Diabetes Mellitus

1. The study presents limited results of insufficient qualitative research with only 14 participants in the entire study.
2. The participants characteristics are inadequately presented. There is need to present the mean age / standard deviation or median age and interquartile range.
3. The result column indicating duration of illness is unclear. What illness was considered?
4. A single session of in-dept interview is inadequate to reach remarkable conclusions.
5. Conclusions made are not valid because results are insufficiently presented from inadequate data.
6. The research methods and design have not referenced to justify the use of 14 participants for only a single session. This compromises the integrity of the whole study.
7. The study requires major revisions and may only be accepted when additional information on the methods, the number of sessions of the in-dept interview is more than once.
