# Supplementary material for: Experiences of social isolation among older adults with type 2 diabetes mellitus: A qualitative study
Source: PLoS One. 2026 Aug 10;21(8):e0354450. doi: 10.1371/journal.pone.0354450 (PMC13456196; doi:10.1371/journal.pone.0354450)
Supplement: Supplementary file 5 [file pone.0354450.s005.docx]

21^st^ June 2026

Editor-in-chief

PLOS One

**Reviewer’s Comment post authors response**

This is to affirm that the manuscript titled “Experiences of social isolation among older adults with type 2 diabetes mellitus: a qualitative study” which has been resubmitted to PLOS One for re-review has been re-examined by me. I hereby confirm that the authors have responded satisfactorily to both reviewers comments. I recommend that manuscript be accepted for publication.

Thank you

Dr Babatunde Abdulmajeed Akodu
