# Supplementary material for: Experiences of social isolation among older adults with type 2 diabetes mellitus: A qualitative study
Source: PLoS One. 2026 Aug 10;21(8):e0354450. doi: 10.1371/journal.pone.0354450 (PMC13456196; doi:10.1371/journal.pone.0354450)
Supplement: Peer review history

# Peer review: Decision Letter 0

**DOI:** 10.1371/journal.pone.0354450.r001

**Authors:** André Luis C Ramalho

21 Apr 2026

PONE-D-26-08610

Qualitative Study on Social Isolation Experience of Elderly Patients with Type 2 Diabetes Mellitus

PLOS One

Dear Dr. Hu,

Thank you for submitting your manuscript to PLOS ONE. After careful consideration, we feel that it has merit but does not fully meet PLOS ONE’s publication criteria as it currently stands. Therefore, we invite you to submit a revised version of the manuscript that addresses the points raised during the review process.

==============================

The topic is relevant and timely, particularly in the context of population ageing and chronic disease management, and the manuscript draws on qualitative data that has the potential to provide meaningful insights into patient experiences. However, following careful evaluation of the manuscript and the reviewers’ reports, several substantive concerns have been identified that currently limit its scientific robustness and suitability for publication.

The primary concern relates to the insufficient rigor and transparency of the qualitative methodology. The manuscript does not clearly specify or justify the qualitative approach adopted, and key elements such as participant recruitment, sampling rationale, and the process by which data saturation was achieved are inadequately described. In addition, the analytical procedures lack sufficient detail, particularly regarding coding processes, theme development, and whether multiple researchers were involved in analysis and validation. The manuscript also does not address essential criteria for qualitative rigor—such as credibility, dependability, confirmability, and transferability—nor does it include a reflexivity statement. These omissions need to be addressed comprehensively to meet expected standards.

Regarding the results, while the thematic organization is generally clear and supported by participant quotations, the analysis remains largely descriptive. Greater analytical depth is required to move beyond reporting experiences toward interpreting their meaning within a conceptual or theoretical framework. There is also some overlap between themes and redundancy in the presentation, which reduces clarity and coherence. Refinement of the thematic structure and more selective use of quotations would strengthen this section.

The discussion section requires substantial revision to enhance its scholarly contribution. At present, it largely reiterates the findings and places disproportionate emphasis on prescriptive recommendations that are not always sufficiently grounded in the data. A more critical and interpretive discussion is needed, including stronger engagement with the international literature and clearer articulation of the study’s contribution. In addition, the manuscript does not adequately address its limitations, particularly those related to sample size, single-site design, and contextual specificity, which are essential for assessing transferability.

In summary, while the manuscript addresses an important topic and is based on potentially valuable qualitative data, it requires significant improvement in methodological rigor, analytical depth, and clarity of reporting.

==============================

Please submit your revised manuscript by Jun 05 2026 11:59PM. If you will need more time than this to complete your revisions, please reply to this message or contact the journal office at plosone@plos.org. When you're ready to submit your revision, log on to https://www.editorialmanager.com/pone/ and select the 'Submissions Needing Revision' folder to locate your manuscript file.

Please include the following items when submitting your revised manuscript:

- A letter that responds to each point raised by the academic editor and reviewer(s). You should upload this letter as a separate file labeled 'Response to Reviewers'.
- A marked-up copy of your manuscript that highlights changes made to the original version. You should upload this as a separate file labeled 'Revised Manuscript with Track Changes'.
- An unmarked version of your revised paper without tracked changes. You should upload this as a separate file labeled 'Manuscript'.

If you would like to make changes to your financial disclosure, please include your updated statement in your cover letter. Guidelines for resubmitting your figure files are available below the reviewer comments at the end of this letter.

If applicable, we recommend that you deposit your laboratory protocols in protocols.io to enhance the reproducibility of your results. Protocols.io assigns your protocol its own identifier (DOI) so that it can be cited independently in the future. For instructions see: https://journals.plos.org/plosone/s/submission-guidelines#loc-laboratory-protocols. Additionally, PLOS ONE offers an option for publishing peer-reviewed Lab Protocol articles, which describe protocols hosted on protocols.io. Read more information on sharing protocols at https://plos.org/protocols?utm_medium=editorial-email&utm_source=authorletters&utm_campaign=protocols.

As the corresponding author, your ORCID iD is verified in the submission system and will appear in the published article. PLOS supports the use of ORCID, and we encourage all coauthors to register for an ORCID iD and use it as well. Please encourage your coauthors to verify their ORCID iD within the submission system before final acceptance, as unverified ORCID iDs will not appear in the published article. *Only*  the individual author can complete the verification step; PLOS staff *cannot*  verify ORCID iDs on behalf of authors.

We look forward to receiving your revised manuscript.

Kind regards,

André Luis C Ramalho, PhD

Academic Editor

PLOS One

**Journal Requirements:**

1. When submitting your revision, we need you to address these additional requirements.

Please ensure that your manuscript meets PLOS ONE's style requirements, including those for file naming. The PLOS ONE style templates can be found at

https://journals.plos.org/plosone/s/file?id=wjVg/PLOSOne_formatting_sample_main_body.pdf and

https://journals.plos.org/plosone/s/file?id=ba62/PLOSOne_formatting_sample_title_authors_affiliations.pdf

2. The ethical approval number(s) listed in the manuscript and submission metadata does not match the approval number on the ethical approval document you provided. Please ensure that all approval numbers are correct.

3. Thank you for stating the following in the Acknowledgments Section of your manuscript:

This research received no specific grant from any funding agency in the public, commercial, or not-for-profit sectors.

We note that you have provided funding information that is not currently declared in your Funding Statement. However, funding information should not appear in the Acknowledgments section or other areas of your manuscript. We will only publish funding information present in the Funding Statement section of the online submission form.

Please remove any funding-related text from the manuscript and let us know how you would like to update your Funding Statement. Currently, your Funding Statement reads as follows:

The author(s) received no specific funding for this work.

Please include your amended statements within your cover letter; we will change the online submission form on your behalf.

4. We note that your Data Availability Statement is currently as follows: All relevant data are within the manuscript and its Supporting Information files.

Please confirm at this time whether or not your submission contains all raw data required to replicate the results of your study. Authors must share the “minimal data set” for their submission. PLOS defines the minimal data set to consist of the data required to replicate all study findings reported in the article, as well as related metadata and methods (https://journals.plos.org/plosone/s/data-availability#loc-minimal-data-set-definition).

For example, authors should submit the following data:

- The values behind the means, standard deviations and other measures reported;

- The values used to build graphs;

- The points extracted from images for analysis.

Authors do not need to submit their entire data set if only a portion of the data was used in the reported study.

If your submission does not contain these data, please either upload them as Supporting Information files or deposit them to a stable, public repository and provide us with the relevant URLs, DOIs, or accession numbers. For a list of recommended repositories, please see https://journals.plos.org/plosone/s/recommended-repositories.

If there are ethical or legal restrictions on sharing a de-identified data set, please explain them in detail (e.g., data contain potentially sensitive information, data are owned by a third-party organization, etc.) and who has imposed them (e.g., an ethics committee). Please also provide contact information for a data access committee, ethics committee, or other institutional body to which data requests may be sent. If data are owned by a third party, please indicate how others may request data access.

5. When completing the data availability statement of the submission form, you indicated that you will make your data available on acceptance. We strongly recommend all authors decide on a data sharing plan before acceptance, as the process can be lengthy and hold up publication timelines. Please note that, though access restrictions are acceptable now, your entire data will need to be made freely accessible if your manuscript is accepted for publication. This policy applies to all data except where public deposition would breach compliance with the protocol approved by your research ethics board. If you are unable to adhere to our open data policy, please kindly revise your statement to explain your reasoning and we will seek the editor's input on an exemption. Please be assured that, once you have provided your new statement, the assessment of your exemption will not hold up the peer review process.

6. Please include your full ethics statement in the ‘Methods’ section of your manuscript file. In your statement, please include the full name of the IRB or ethics committee who approved or waived your study, as well as whether or not you obtained informed written or verbal consent. If consent was waived for your study, please include this information in your statement as well.

7. If the reviewer comments include a recommendation to cite specific previously published works, please review and evaluate these publications to determine whether they are relevant and should be cited. There is no requirement to cite these works unless the editor has indicated otherwise.

[Note: HTML markup is below. Please do not edit.]

Reviewers' comments:

Reviewer's Responses to Questions

**Comments to the Author**

1. Is the manuscript technically sound, and do the data support the conclusions?

The manuscript must describe a technically sound piece of scientific research with data that supports the conclusions. Experiments must have been conducted rigorously, with appropriate controls, replication, and sample sizes. The conclusions must be drawn appropriately based on the data presented.

Reviewer #1: Partly

Reviewer #2: No

**********

2. Has the statistical analysis been performed appropriately and rigorously?

Reviewer #1: N/A

Reviewer #2: No

**********

3. Have the authors made all data underlying the findings in their manuscript fully available?

The PLOS Data policy requires authors to make all data underlying the findings described in their manuscript fully available without restriction, with rare exception (please refer to the Data Availability Statement in the manuscript PDF file). The data should be provided as part of the manuscript or its supporting information, or deposited to a public repository. For example, in addition to summary statistics, the data points behind means, medians and variance measures should be available. If there are restrictions on publicly sharing data—e.g. participant privacy or use of data from a third party—those must be specified.

Reviewer #1: Yes

Reviewer #2: Yes

**********

4. Is the manuscript presented in an intelligible fashion and written in standard English?

PLOS ONE does not copyedit accepted manuscripts, so the language in submitted articles must be clear, correct, and unambiguous. Any typographical or grammatical errors should be corrected at revision, so please note any specific errors here.

Reviewer #1: No

Reviewer #2: Yes

**********

5. Review Comments to the Author

Please use the space provided to explain your answers to the questions above. You may also include additional comments for the author, including concerns about dual publication, research ethics, or publication ethics. (Please upload your review as an attachment if it exceeds 20,000 characters)

**Reviewer #1:**  The manuscript addresses an important topic but shows consistent weaknesses across all sections. The introduction lacks clarity, logical flow, and a clearly articulated research gap with several language and structural issues. The methodology provides a basic qualitative framework but lacks rigor with insufficient detail on sampling, data saturation, analytical procedures, and trustworthiness criteria. The results are well-organized and supported by rich participant quotes, yet remain largely descriptive with overlap between themes and limited analytical depth. The discussion aligns broadly with the findings but mainly restates results, overemphasizes recommendations and lacks critical engagement with literature and study limitations. Overall, the study requires substantial revision to improve coherence, methodological transparency and analytical rigor. I have provided few points below to improve the article for future correspondence. But this time I am unable to accept its publication due to the standard of the esteem journal.

Introduction:

The introduction addresses a relevant and important topic (T2DM, ageing, and social isolation). However, it has major issues in clarity, structure, language and logical flow. It currently reads as fragmented and partially incomplete and requires substantial revision before being suitable for publication. It needs deep background check.

• Lack of clarity and coherence: The introduction contains grammatical errors, incomplete sentences and unclear phrasing, making it difficult to follow.

• Poor structure and organization: Ideas are presented in a disjointed manner, with no logical progression from background to research gap and study aim.

• Weak research gap and justification: The need for the study- especially the qualitative approach is not clearly or convincingly explained.

• Inadequate use of literature: Citations are poorly integrated and some claims are oversimplified or lack proper contextual explanation.

• Conceptual and scientific issues: Definitions (e.g., social isolation) are somewhat inaccurate or oversimplified and causal relationships are presented too simplistically.

• Language and readability problems: The text requires substantial editing for grammar, conciseness and academic tone.

Methodology:

Methodology demonstrates a basic qualitative framework; however, it lacks sufficient rigor, transparency and detail. Key aspects such as sampling justification, data saturation, analytic procedures and trustworthiness criteria are inadequately addressed. In tables no mention of units i.e. Age (years/months/days???), Duration of illness (years/months/days???). These are very basic. Substantial revision is required to meet qualitative research reporting standards.

• Unclear study design: The qualitative approach is not well specified or justified and the choice of design is inadequately explained.

• Sampling limitations: Purposive sampling is mentioned but recruitment procedures, sampling rationale, and evidence of data saturation are insufficiently described; single-site sampling limits generalizability.

• Insufficient detail in data collection: Interview procedures lack depth with no discussion of researcher reflexivity, interviewer role or adequacy of interview duration.

• Weak rigor and quality assurance: Key criteria for qualitative rigor (credibility, dependability, confirmability, transferability) are not addressed.

• Inadequate data analysis description: The coding and thematic analysis process lacks transparency, with no detail on coding framework, inter-coder agreement, or theme development.

• Ethical and reporting gaps: Ethics approval is not reported and the methodology does not adhere to established qualitative reporting standards.

Results:

Results part partially align with the study objective of exploring social isolation among elderly patients with type 2 diabetes, as they present relevant experiences related to psychological distress, relational dynamics and environmental barriers. However, this alignment is not consistently strong or explicit. The central concept of social isolation is not systematically integrated across all themes and some findings (e.g., environmental or healthcare system issues) appear indirectly related rather than clearly tied to the core objective. Additionally, the lack of deeper analytical interpretation and weak link to the guiding theoretical framework limit the extent to which the findings fully address and advance the stated research aim. There is overlap between themes, limited integration with the theoretical framework and issues with redundancy and verbosity. Greater clarity, conceptual refinement, and analytical interpretation are required to strengthen the findings.

• Thematic structure issues: Although themes and subthemes are clearly presented, there is overlap between categories and insufficient justification for the thematic framework.

• Limited analytical depth: The findings are largely descriptive with minimal interpretation or higher-level conceptual analysis.

• Quotation overuse: Participant quotes are rich but often too lengthy and repetitive, reducing clarity and impact. Please learn how quotes are presented in academic journal. Very poor presentation.

• Weak integration with theory and objectives: The results are not consistently linked to the stated theoretical framework (social ecological theory) or clearly anchored to the core concept of social isolation.

• Redundancy and lack of rigor: Repetition of similar ideas, absence of thematic prevalence indicators and limited transparency in how findings were derived weaken the rigor.

Discussion:

Discussion demonstrates a clear effort to organise findings within a socio-ecological framework and maintains thematic consistency with the results. However, it falls short in delivering critical, in-depth interpretation and scholarly engagement. The section reads more as a set of practical recommendations than a rigorous academic discussion, with insufficient attention to limitations, contextualization and theoretical advancement. Substantial revision is needed to strengthen analytical depth, balance, and scientific contribution.

• Limited analytical depth: Discussion largely restates the results using theoretical language, with insufficient deeper interpretation or exploration of underlying mechanisms.

• Weak engagement with literature: Existing studies are cited mainly for support, with little critical comparison or positioning of findings within broader research.

• Overemphasis on recommendations: A substantial portion focuses on intervention strategies which are often overly prescriptive and not fully grounded in the data.

• Lack of methodological reflection: There is no discussion of study limitations, context or transferability, leading to potential overgeneralization of findings.

• Imbalance and structure issues: The section is uneven, with minimal interpretive discussion and extensive policy-like recommendations, reducing academic rigor.

• Underutilization of qualitative insights: The richness of the data is not fully translated into higher-level conceptual or theoretical contributions.

**Reviewer #2:**  1. The study presents limited results of insufficient qualitative research with only 14 participants in the entire study.

2. The participants characteristics are inadequately presented. There is need to present the mean age / standard deviation or median age and interquartile range.

3. The result column indicating duration of illness is unclear. What illness was considered?

4. A single session of in-dept interview is inadequate to reach remarkable conclusions.

5. Conclusions made are not valid because results are insufficiently presented from inadequate data.

6. The research methods and design have not referenced to justify the use of 14 participants for only a single session. This compromises the integrity of the whole study.

7. The study requires major revisions and may only be accepted when additional information on the methods, the number of sessions of the in-dept interview is more than once.

**********

6. PLOS authors have the option to publish the peer review history of their article (what does this mean?). If published, this will include your full peer review and any attached files.

If you choose “no”, your identity will remain anonymous but your review may still be made public.

**Do you want your identity to be public for this peer review?**  For information about this choice, including consent withdrawal, please see our Privacy Policy.

Reviewer #1: **Yes:**  Dr. Ipsita Basu.

Researcher

INRCA - IRCCS l Italy

(National Institute of Health and Science on Ageing)

Reviewer #2: **Yes:**  Babatunde Abdulmajeed Akodu

**********

[NOTE: If reviewer comments were submitted as an attachment file, they will be attached to this email and accessible via the submission site. Please log into your account, locate the manuscript record, and check for the action link "View Attachments". If this link does not appear, there are no attachment files.]

To ensure your figures meet our technical requirements, please review our figure guidelines: https://journals.plos.org/plosone/s/figures

You may also use PLOS’s free figure tool, NAAS, to help you prepare publication quality figures: https://journals.plos.org/plosone/s/figures#loc-tools-for-figure-preparation.

NAAS will assess whether your figures meet our technical requirements by comparing each figure against our figure specifications.

Attachment Submitted filename: Social isolation in elder DM Plos One.pdf

Attachment Submitted filename: Qualitative Study on Social Isolation Diabetes comments.docx

# Author comment: Author response to Decision Letter 1

**DOI:** 10.1371/journal.pone.0354450.r002

29 May 2026

Thank you very much for your careful evaluation of our manuscript entitled “Qualitative Study on Social Isolation Experience of Elderly Patients with Type 2 Diabetes Mellitus.” We sincerely appreciate the time and effort you have devoted to reviewing our work and providing valuable comments. Your constructive suggestions have been extremely helpful in improving the quality of our manuscript.

We have carefully considered all comments and revised the manuscript accordingly.

Attachment Submitted filename: Response to Reviewers.docx

# Peer review: Decision Letter 1

**DOI:** 10.1371/journal.pone.0354450.r003

**Authors:** André Luis C Ramalho

8 Jul 2026

Experiences of social isolation among older adults with type 2 diabetes mellitus: a qualitative study

PONE-D-26-08610R1

Dear Dr. Li,

We’re pleased to inform you that your manuscript has been judged scientifically suitable for publication and will be formally accepted for publication once it meets all outstanding technical requirements.

Within one week, you’ll receive an e-mail detailing the required amendments. When these have been addressed, you’ll receive a formal acceptance letter and your manuscript will be scheduled for publication.

An invoice will be generated when your article is formally accepted. Please note, if your institution has a publishing partnership with PLOS and your article meets the relevant criteria, all or part of your publication costs will be covered. Please make sure your user information is up-to-date by logging into Editorial Manager at Editorial Manager® and clicking the ‘Update My Information' link at the top of the page. For questions related to billing, please contact billing support.

If your institution or institutions have a press office, please notify them about your upcoming paper to help maximize its impact. If they’ll be preparing press materials, please inform our press team as soon as possible -- no later than 48 hours after receiving the formal acceptance. Your manuscript will remain under strict press embargo until 2 pm Eastern Time on the date of publication. For more information, please contact onepress@plos.org.

Kind regards,

André Luis C Ramalho, PhD

Academic Editor

PLOS One

Reviewers' comments:

Reviewer's Responses to Questions

**Comments to the Author**

1. If the authors have adequately addressed your comments raised in a previous round of review and you feel that this manuscript is now acceptable for publication, you may indicate that here to bypass the “Comments to the Author” section, enter your conflict of interest statement in the “Confidential to Editor” section, and submit your "Accept" recommendation.

Reviewer #2: All comments have been addressed

**********

2. Is the manuscript technically sound, and do the data support the conclusions?

The manuscript must describe a technically sound piece of scientific research with data that supports the conclusions. Experiments must have been conducted rigorously, with appropriate controls, replication, and sample sizes. The conclusions must be drawn appropriately based on the data presented.

Reviewer #2: Yes

**********

3. Has the statistical analysis been performed appropriately and rigorously?

Reviewer #2: Yes

**********

4. Have the authors made all data underlying the findings in their manuscript fully available?

The PLOS Data policy requires authors to make all data underlying the findings described in their manuscript fully available without restriction, with rare exception (please refer to the Data Availability Statement in the manuscript PDF file). The data should be provided as part of the manuscript or its supporting information, or deposited to a public repository. For example, in addition to summary statistics, the data points behind means, medians and variance measures should be available. If there are restrictions on publicly sharing data—e.g. participant privacy or use of data from a third party—those must be specified.

Reviewer #2: Yes

**********

5. Is the manuscript presented in an intelligible fashion and written in standard English?

PLOS ONE does not copyedit accepted manuscripts, so the language in submitted articles must be clear, correct, and unambiguous. Any typographical or grammatical errors should be corrected at revision, so please note any specific errors here.

Reviewer #2: Yes

**********

6. Review Comments to the Author

Please use the space provided to explain your answers to the questions above. You may also include additional comments for the author, including concerns about dual publication, research ethics, or publication ethics. (Please upload your review as an attachment if it exceeds 20,000 characters)

Reviewer #2: Authors have satisfactory responded to the queries raised in the initial submission.

They have improved the table title and participants characteristics. Authors have provided more details in the materials and methods section with more clarity. The discussion is now well compared and contrasted with past studies with reasons for similarities and differences.

This is to affirm that the manuscript titled “Experiences of social isolation among older adults with type 2 diabetes mellitus: a qualitative study” which has been resubmitted to PLOS One for re-review has been re-examined by me. I hereby confirm that the authors have responded satisfactorily to both reviewers comments. I recommend that manuscript be accepted for publication.

**********

7. PLOS authors have the option to publish the peer review history of their article (what does this mean?). If published, this will include your full peer review and any attached files.

If you choose “no”, your identity will remain anonymous but your review may still be made public.

**Do you want your identity to be public for this peer review?**  For information about this choice, including consent withdrawal, please see our Privacy Policy.

Reviewer #2: **Yes:**  Babatunde Abdulmajeed Akodu

**********

Attachment Submitted filename: Reviwers comment for PLOS One 2026.docx

# Editor report: Acceptance letter

**DOI:** 10.1371/journal.pone.0354450.r004

**Authors:** André Luis C Ramalho

PONE-D-26-08610R1

PLOS One

Dear Dr. Li,

I'm pleased to inform you that your manuscript has been deemed suitable for publication in PLOS One. Congratulations! Your manuscript is now being handed over to our production team.

At this stage, our production department will prepare your paper for publication. This includes ensuring the following:

* All references, tables, and figures are properly cited

* All relevant supporting information is included in the manuscript submission,

* There are no issues that prevent the paper from being properly typeset

You will receive further instructions from the production team, including instructions on how to review your proof when it is ready. Please keep in mind that we are working through a large volume of accepted articles, so please give us a few days to review your paper and let you know the next and final steps.

Lastly, if your institution or institutions have a press office, please let them know about your upcoming paper now to help maximize its impact. If they'll be preparing press materials, please inform our press team within the next 48 hours. Your manuscript will remain under strict press embargo until 2 pm Eastern Time on the date of publication. For more information, please contact onepress@plos.org.

You will receive an invoice from PLOS for your publication fee after your manuscript has reached the completed accept phase. If you receive an email requesting payment before acceptance or for any other service, this may be a phishing scheme. Learn how to identify phishing emails and protect your accounts at https://explore.plos.org/phishing.

If we can help with anything else, please email us at customercare@plos.org.

Thank you for submitting your work to PLOS ONE and supporting open access.

Kind regards,

PLOS ONE Editorial Office Staff

on behalf of

Prof. Dr. André Luis C Ramalho

Academic Editor

PLOS One
